# Genomic characteristics of triple negative apocrine carcinoma: a comparison to triple negative breast cancer

**DOI:** 10.1038/s12276-023-01030-z

**Published:** 2023-07-03

**Authors:** Ji-Yeon Kim, Sabin Park, Eun Yoon Cho, Jeong Eon Lee, Hae Hyun Jung, Byung Joo Chae, Seok Won Kim, Seok Jin Nam, Soo Youn Cho, Yeon Hee Park, Jin Seok Ahn, Semin Lee, Young-Hyuck Im

**Affiliations:** 1 Division of Hematology-Oncology, Department of Internal Medicine, Samsung Medical Center, Sungkyunkwan University School of Medicine, Seoul, 06351 Republic of Korea; 2 Biomedical Research Institute, Samsung Medical Center, Seoul, 06351 Republic of Korea; 3 Department of Health Sciences and Technology, Samsung Advanced Institute for Health Sciences and Technology, Seoul, 06351 Republic of Korea; 4 Department of Biomedical Engineering, College of Information-Bio Convergence Engineering, Ulsan National Institute of Science and Technology, Ulsan, 44919 Republic of Korea; 5 Department of Pathology, Samsung Medical Center, Sungkyunkwan University School of Medicine, Seoul, 06351 Republic of Korea; 6 Department of Surgery, Samsung Medical Center, Sungkyunkwan University School of Medicine, Seoul, 06351 Republic of Korea

**Keywords:** Genetics research, Translational research, Breast cancer, Cancer genomics

## Abstract

Apocrine carcinoma is a rare breast cancer subtype. As such, the genomic characteristics of apocrine carcinoma with triple negative immunohistochemical results (TNAC), which has been treated as triple negative breast cancer (TNBC), have not been revealed. In this study, we evaluated the genomic characteristics of TNAC compared to TNBC with low Ki-67 (LK-TNBC). In the genetic analysis of 73 TNACs and 32 LK-TNBCs, the most frequently mutated driver gene in TNAC was *TP53* (16/56, 28.6%), followed by *PIK3CA* (9/56, 16.1%), *ZNF717* (8/56, 14.3%), and *PIK3R1* (6/56, 10.71%). Mutational signature analysis showed enrichment of defective DNA mismatch repair (MMR)-related signatures (SBS6 and SBS21) and the SBS5 signature in TNAC, whereas an APOBEC activity-associated mutational signature (SBS13) was more prominent in LK-TNBC (Student’s *t* test, *p* < 0.05). In intrinsic subtyping, 38.4% of TNACs were classified as luminal A, 27.4% as luminal B, 26.0% as HER2-enriched (HER2-E), 2.7% as basal, and 5.5% as normal-like. The basal subtype was the most dominant subtype (43.8%) in LK-TNBC (*p* < 0.001), followed by luminal B (21.9%), HER2-E (21.9%), and luminal A (12.5%). In the survival analysis, TNAC had a five-year disease-free survival (DFS) rate of 92.2% compared to 59.1% for LK-TNBC (*P* = 0.001) and a five-year overall survival (OS) rate of 95.3% compared to 74.6% for LK-TNBC (*P* = 0.0099). TNAC has different genetic characteristics and better survival outcomes than LK-TNBC. In particular, normal-like and luminal A subtypes in TNAC have much better DFS and OS than other intrinsic subtypes. Our findings are expected to impact medical practice for patients diagnosed with TNAC.

## Breast cancer: Genomic characteristics of rare subtype examined

A comparative study reveals the distinctive genomic and clinical characteristics of a rare form of breast cancer, with implications for treatment. Apocrine carcinoma accounts for 1–4 percent of breast cancer cases. The molecular characteristics of a subtype known as triple negative apocrine carcinoma (TNAC) remain unclear. Ji-Yeon Kim at Sungkyunkwan University School of Medicine, Seoul, South Korea, and co-workers conducted genomic analysis on tumor samples taken from 73 TNAC patients. They compared the results to samples from 32 triple negative breast cancer (TNBC) in which the low expression of Ki-67, a marker protein for cancer proliferation, was similar to the TNAC patients. TNAC patients had different genetic mutations and a better survival outcome than TNBC patients with low Ki-67. Therefore, early-stage TNAC may not require chemotherapy after surgery. Further research into TNAC genomic characteristics is warranted.

## Introduction

Apocrine carcinoma is a rare breast cancer (BC) subtype accounting for 1–4% of all BCs^1^. The 2019 WHO classification of breast tumors defined apocrine carcinoma as a distinct, special type of BC characterized by large round nuclei with plump, eosinophilic, granular, and sharp-bordered cytoplasm^2^. Apocrine carcinomas frequently express androgen receptor (AR) but are estrogen receptor (ER) and progesterone receptor (PgR) negative^3^. Therefore, apocrine carcinomas may be divided into two BC subtypes based on human epidermal growth factor receptor-2 (HER2) status: triple negative breast cancer (TNBC) and HER2-positive BC^1,3^.

TNBC is ER- and PgR-negative, does not overexpress HER2, and is usually included in the “basal-like BC” group according to the intrinsic subtype^4–7^. TNBCs account for 15% of all BCs, and they generally have poor clinical outcomes^4,7^. While this aggressive phenotype is common, there is a small group of TNBCs with favorable clinical outcomes showing a low risk of recurrence and death^8^. Low-risk TNBCs comprise salivary gland-type BC, TNBC with high tumor-infiltrating lymphocytes (TILs), and carcinoma with apocrine differentiation^9^. Apocrine carcinoma with triple negative immunohistochemical results (TNAC) has a low Ki-67 labeling index and is very different from most aggressive TNBCs, although the underlying molecular characteristics of apocrine breast cancer have been poorly studied^10^.

Previous study has demonstrated that TNAC has a poorer response to (neo)adjuvant chemotherapy than other non-apocrine TNBC^11^. However, survival analyses have shown discordant results. One previous study showed similar survival outcomes in TNAC and TNBC, whereas another reported poor prognosis in TNAC, and yet another reported better survival outcome in TNAC than in TNBC^9,12,13^. Therefore, comprehensive genomic profiling of TNAC could provide a detailed understanding of the molecular biology and prognosis and suggest potential therapeutic targets for individualized treatment. In this study, we evaluated the genomic and clinical characteristics of TNAC and compared them to those of TNBC with low Ki-67 expression.

## Methods

### Patients

Tumors were selected from 73 TNAC patients who underwent curative surgery at Samsung Medical Center, Seoul, Korea. In addition, we selected 32 TNBCs that had Ki-67 levels similar to those of TNACs from patients who underwent curative surgery. Independent pathological review was performed to determine the apocrine type and triple negativity of BC. The Institutional Review Board of Samsung Medical Center approved the study protocol (IRB No: 2020-05-159).

### Whole-exome sequencing

#### Extraction of DNA

DNA was extracted from formalin-fixed paraffin-embedded (FFPE) blocks or fresh frozen (FF) tissues. A skilled pathologist reviewed hematoxylin and eosin (H&E)-stained sections from the FFPE blocks and outlined areas containing representative invasive breast carcinoma on the slide. FFPE slides were manually microdissected to maximize tumor purity. Genomic DNA was extracted using the ReliaPrep FFPE gDNA Miniprep System (Promega), the QIAamp DNA Mini Kit (Qiagen), and the QIAamp DNA Blood Mini Kit (Qiagen) for FFPE, FF tissues, and buffy coats, respectively.

#### Sequencing: Illumina NovaSeq6000 platform

For generation of standard exome capture libraries, we used 1 µg of input gDNA and the Agilent SureSelect Target Enrichment protocol for generating an Illumina paired-end sequencing library. In all cases, the SureSelect Human All Exon V6 probe set was used. DNA concentration and DNA quality were measured by PicoGreen and agarose gel electrophoresis. We used 1 μg of each cell line’s genomic DNA diluted in EB buffer and sheared to a target peak size of 150–200 bp using the Covaris LE220 focused-ultrasonicator (Covaris, Woburn, MA) according to the manufacturer’s recommendations. An 8 microTUBE Strip was loaded into the tube holder of the ultrasonicator, and DNA was sheared using the following settings: mode, frequency sweeping; duty cycle, 10%; intensity, 5; cycles per burst, 200; duration, 60 s × 6 cycles; temperature, 4–7 °C. The fragmented DNA was repaired, an ‘A’ was ligated to the 3′ end, and Agilent adapters were ligated to the fragments.

Once ligation had been assessed, the adapter ligated product was PCR amplified. The final purified product was then quantified using the TapeStation DNA screentape D1000 (Agilent). For exome capture, 250 ng of DNA library was mixed with hybridization buffer, blocking mixture, RNase block and 5 µl of SureSelect all exon capture library, according to the standard Agilent SureSelect Target Enrichment protocol. Hybridization to the capture baits was conducted at 65 °C using a heated thermal cycler lid option at 105 °C for 24 h on a PCR machine. The captured DNA was then washed and amplified. The final purified product was then quantified using qPCR according to the qPCR Quantification Protocol Guide (KAPA Library Quantification kit for Illumina Sequencing platforms) and qualified using the TapeStation DNA screentape D1000 (Agilent). Indexed libraries were then sequenced using the NovaSeq6000 platform (Illumina, San Diego, USA) by Macrogen Incorporated.

#### Sequence alignment

Paired-end reads were aligned to the GRCh38 human reference genome using the Burrows Wheeler aligner^14^ (BWA v0.7.17), and BAM (binary alignment/map) files were produced for each sample. Local realignment and quality score recalibration processes were performed using Genome Analysis Toolkit^15^ (GATK v4.2). Sequencing quality was evaluated using fastQC and the Picard CollectMultipleMetrics tool (https://broadinstitute.github.io/picard/).

#### Somatic point mutation detection

The TNAC dataset consists of 54 duplicated tumor samples and 2 nonduplicated tumor samples (C009, C012) with matched normal controls. The LK-TNBC dataset consists of 27 duplicated tumor samples with matched normal controls.

Mutations were called for each duplicate tumor sample with matched control. For nonduplicated tumor samples (C009, C012), fastq files were split randomly into two using the SeqKit tool. Somatic point mutations and short indels were called using MuTect2^16^ from GATK v4.2. The filtering process was performed to reduce sequencing or data processing errors from FFPE breast samples^17^. We selected only variants detected in both duplicate bam files for one sample. To detect significantly mutated driver genes, the dNdScv algorithm^18^ was applied. We selected variants with p values less than 0.001 in TNAC and LK-TNBC.

TCGA luminal A and TCGA TNBC somatic mutations were downloaded from the GDC data portal^5^.

#### Mutational signature analysis

Mutational signature analysis was performed with the deconstructSigs^19^ R package. It calculates the proportion of 30 COSMIC signatures for each sample. We selected signatures that showed a significant difference in proportion between TNAC and LK-TNBC, and between hypermutated samples and nonhypermutated samples in TNAC. Signatures that showed no significant difference when outliers were removed were excluded. Significance was calculated by Student’s *t* test.

To examine DNA mismatch repair (MMR)-related signatures in more detail, we assessed the frequency of MMR-related gene mutations. A total of 13 MMR-related genes, including MSH2, MSH3, MSH4, MSH5, MSH6, MLH1, MLH3, PMS1, PMS2, POLD1, POLB, POLE, and POLG, were used for analysis.

#### Somatic copy number alteration analysis

CNVkit v0.9.9 was used to call somatic copy number alterations (SCNAs) on whole-exome sequencing data. In the results, log_2_ depth ratios greater than 0.2 were considered to indicate copy number gain, and ratios less than −0.2 were considered to indicate copy number loss.

The significance of somatic copy number alterations was evaluated using GISTIC2.0^20^ (Version 2.0.23), which deconstructs SCNAs into broad and focal events. We added the ranges of 1,000,000 paddings considering information on centromere and telomere positions from UCSC hg38. Cancer-related gene sets used for matching the genes covering the resulting peaks were downloaded from the COSMIC Cancer Gene Census (CGC) database.

### Whole-transcriptome sequencing

#### Extraction of RNA

RNA was extracted from formalin-fixed paraffin-embedded (FFPE) blocks or fresh frozen (FF) tissues. A skilled pathologist reviewed hematoxylin and eosin (H&E)-stained sections from the FFPE blocks and outlined areas containing representative invasive breast carcinoma on the slide. FFPE slides were manually microdissected to maximize tumor purity.

Total RNA was extracted using the ReliaPrep FFPE Total RNA Miniprep System (Promega) and RNeasy Mini Kit (Qiagen) for FFPE and FF specimens, respectively, according to the manufacturer’s protocol. Nucleic acid yield and purity were assessed using a NanoDrop ND-1000 Spectrophotometer (NanoDrop Technologies, Thermo-Fisher Scientific, MA, USA).

#### Sequencing: Illumina NovaSeq6000 platform

The total RNA concentration was calculated by Quant-IT RiboGreen (Invitrogen). To determine the DV200 (% of RNA fragments >200 bp) value, samples were run on the TapeStation RNA screentape (Agilent). Then, 100 ng of total RNA was subjected to sequencing library construction using a TruSeq RNA Access library prep kit (Illumina, San Diego, CA, USA) according to the manufacturer’s protocols. Briefly, the total RNA was fragmented into small pieces using divalent cations under elevated temperature. The cleaved RNA fragments were copied into first-strand cDNA using SuperScript II reverse transcriptase (Invitrogen, #18064014) and random primers. This was followed by second-strand cDNA synthesis using DNA polymerase I, RNase H, and dUTP. These cDNA fragments then went through an end-repair process; single ‘A’ bases were added, and the adapters were ligated. The products were purified and enriched via PCR to create cDNA libraries. All libraries were normalized, and six libraries were pooled into a single hybridization/capture reaction. Pooled libraries were incubated with a cocktail of biotinylated oligos corresponding to coding regions of the genome. Targeted library molecules were captured via hybridized biotinylated oligo probes using streptavidin-conjugated beads. After two rounds of hybridization/capture reactions, the enriched library molecules were subjected to a second round of PCR amplification. The captured libraries were quantified using KAPA Library Quantification kits for Illumina Sequencing platforms according to the qPCR Quantification Protocol Guide (KAPA BIOSYSTEMS, #KK4854) and qualified using the TapeStation D1000 ScreenTape (Agilent Technologies, # 5067-5582). Indexed libraries were submitted to an Illumina NovaSeq6000 (Illumina, Inc.), and paired-end (2×100 bp) sequencing was performed by Macrogen Incorporated.

#### Sequence alignment and quantification

Whole transcriptome sequencing reads were mapped to the GRCh38 human reference genome from GENCODE v38 using STAR v2.7.9a. Fragments per kilobase of exon model per million mapped fragments (FPKM) values were obtained using RSEM v1.3.2. Sequencing quality was evaluated using fastQC and FastQ Screen 0.14.0.

#### Batch correction

Batch effects between FF and FFPE samples were corrected using ComBat-seq^21^, and then FPKM normalization was performed using the convertCounts function in DGEobj.utils of the R package. To conduct batch correction between the TCGA cohort and our batch-corrected dataset, the ComBat^22^ function in the sva package was used. The expression profiles were transformed as follows: log_2_(FPKM + 1).

#### Intrinsic subtype prediction

Intrinsic subtypes of each sample were determined by the ‘molecular.subtyping’ function in the genefu^23^ bioconductor package (v2.26.0). The subtyping classification model was ‘pam50’, which identifies breast cancer molecular subtypes as luminal A, luminal B, HER2-enriched, basal, and normal-like subtypes based on the PAM50 genes. Hierarchical clustering based on the Ward D2 method with a Euclidean distance measure was used to co-cluster our cohort and the TCGA BRCA cohort based on the PAM50 genes. We transformed the expression matrix by log_2_ (FPKM + 1) and then used pheatmap v1.0.12 in the R package to scale the results.

#### Lehmann subtype prediction

Lehmann subtyping^24^ was performed using a web-based subtyping tool, TNBCtype^25^. Input samples were assigned to one of the TNBC subtypes of BL1 (basal-like 1), BL2 (basal-like 2), IM (immunomodulatory), M (mesenchymal), MSL (mesenchymal stem-like), LAR (luminal androgen receptor), and UNS (unstable). Genes with a mean expression values greater than 5 FPKM across all samples were used as input data. If the number of genes which had higher level of gene expressions compared with that of ESR1 was less than 75% of samples, the sample was classified as ER-positive rather than TNBC and was excluded. Five samples in our dataset (C025, C052, C062, K002, K040) and seven samples of TCGA TNBC data^5^ (TCGA-A2-A0ST-01A, TCGA-AR-A0U1-01A, TCGA-B6-A0IE-01A, TCGA-B6-A0IK-01A, TCGA-B6-A0RG-01A, TCGA-B6-A0RN-01A, TCGA-BH-A1EW-01A) were predicted to be ER-positive and removed before running TNBCtype. These excluded samples were classified as ‘unstable’. Hierarchical clustering based on the Ward D2 method with a Euclidean distance measure was used to co-cluster our cohort and the TCGA TNBC cohort. Genes that distinguished Lehmann subtypes^24^ were selected for clustering. We transformed the expression matrix by log_2_ (FPKM + 1) and then used pheatmap v1.0.12 in the R package to scale the results.

#### Burstein and FUSCC subtyping

In Burstein subtyping^26^, hierarchical clustering based on the Ward D2 method with a Euclidean distance measure was used to cluster our cohort into Burstein subtypes of luminal androgen receptor (LAR), mesenchymal (MES), basal-like immunosuppressed (BLIS), and basal-like immune-activated (BLIA). Fifty-five genes significantly overexpressed in each subtype defined by Burstein et al. ^26^ were selected for clustering to determine the characteristics of TNAC and LK-TNBC. We transformed the expression matrix by log_2_ (FPKM + 1) and then used pheatmap v1.0.12 in the R package to scale the results.

In FUSCC subtyping^27^, hierarchical clustering based on the Ward D2 method with a Euclidean distance measure was used to co-cluster our cohort and the TCGA TNBC cohort to identify the subtype mainly grouped with TNAC and LK-TNBC. The TCGA TNBC cohort annotated with both Lehmann subtype and FUSCC classification was provided by Jiang, Yi-Zhou, et al. ^28^. The top 2000 most variable genes in the TCGA cohort were selected for clustering. We transformed the expression matrix by calculating log_2_ (FPKM + 1) and then used pheatmap v1.0.12 in the R package to scale the results.

#### Differential gene expression (DGE) analysis and enriched pathway analysis

DGE analysis between TNAC and LK-TNBC was performed using DESeq2. Genes with |log_2_-fold change| > 2, *p* value < 0.01, and adjusted *p* value < 0.01 were considered differentially expressed genes (DEGs). We performed gene set enrichment analysis^29^ (GSEA v3.0) to identify pathways enriched in TNAC or LK-TNBC. Pathways with FDR *q*-values less than 0.05 were selected.

DGE analysis was also performed between intrinsic subtypes of TNAC using NOISeqBIO^30^ to compare one subtype to the others. Genes with | log_2_-fold change| > 1 and probability > 0.95 were considered DEGs. Gene set enrichment analysis was performed using Enrichr^31^. Significantly enriched pathways from MSigDB Hallmark 2020, KEGG 2021 Human, and GO Biological Process 2021 were selected (adjusted *p* value < 0.05).

#### Cell type estimation analysis

The abundance of 29 cell types as defined in Wu et al. ^32^ was used for deconvolution analysis of our bulk WTS data. CIBERSORTx^33^ was mainly performed to estimate cell types for each sample, and MuSiC^34^ v0.2.0 and BisqueRNA^35^ v1.0.5 were used to verify the results of CIBERSORTx. Cell types that showed significant differences with at least one of these two additional tools were selected. Significance was calculated by the Wilcoxon signed-rank test.

#### Gene fusion analysis

Arriba^36^ v2.1.0 was used to detect gene fusions and selected those with confidence of ‘high’ or ‘medium’. Fusion genes detected in healthy individuals (*n* = 87)^37^ with a frequency of less than 0.05 were filtered. The difference in the frequency of gene fusion events was compared between TNAC and LK-TNBC and between intrinsic subtypes. Significance was calculated by the Wilcoxon signed-rank test and Kruskal‒Wallis test.

### Statistical analysis

We evaluated the differences between TNAC and Ki-67-matched TNBC (low Ki-67 TNBC; LK-TNBC) using Fisher’s exact test. Disease-free survival (DFS) was defined as the duration of survival without any signs or symptoms of disease after primary treatment. Overall survival (OS) was defined as the duration between curative surgery and death. DFS and OS were analyzed using the Kaplan‒Meier method. Cox proportional hazard regression was used to estimate hazard ratios and 95% confidence intervals (CIs).

## Results

### Baseline characteristics

We described the baseline clinical and pathological characteristics of TNAC and LK-TNBC in Supplementary Table 1. Only stage at diagnosis was different between TNAC and LK-TNBC (*P* = 0.03), while no significant differences were observed in other characteristics, including nuclear grade, histologic grade, Ki-67, and status of (neo)adjuvant treatment.

### Somatic mutations of TNAC

**Fig. 1:**
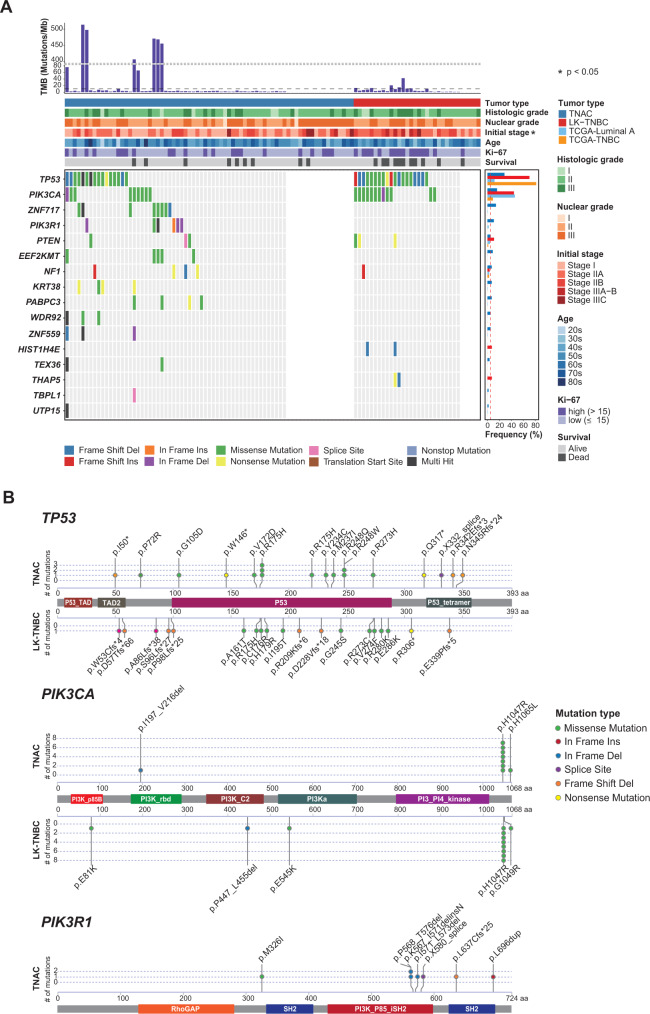
Somatic Point Mutations in TNAC and LK-TNBC. **A** The top 16 significantly mutated genes as determined by the dNdScv algorithm (*p* < 0.001, TNAC: 13 genes, LK-TNBC: 5 genes) in TNAC and LK-TNBC were sorted by their mutation frequency. The right bar plot shows the mutation frequencies of the 16 genes in our cohorts and TCGA cohorts. The red dotted vertical line represents a 5% mutation frequency. The top bar plot indicates tumor mutational burden (TMB), which represents the number of mutations per megabase (Mb) for each sample. The samples with TMB values greater than 10 were considered hypermutated (black dotted horizontal line). The clinical and pathological characteristics were also annotated. The significant differences between TNAC and LK-TNBC according to baseline characteristics were calculated by Fisher’s exact test. **B** Schematic representation of somatic point mutations in *TP53*, *PIK3CA*, and *PIK3R1* in our TNAC and LK-TNBC cohorts.

We identified 18,747 and 2097 nonsynonymous somatic point mutations from TNAC and LK-TNBC WES data, respectively. Somatic point mutations were detected using Mutect2 with a filtering process as described in the Methods. The most frequently mutated driver gene in TNAC was *TP53* (16/56, 28.6%), followed by *PIK3CA* (9/56, 16.1%), *ZNF717* (8/56, 14.3%), and *PIK3R1* (6/56, 10.71%) (Fig. 1A). In our cohort, the incidence of *TP53* mutations was lower in TNAC than in both the LK-TNBC and TCGA-TNBC datasets, in line with previous studies^38,39^. However, *PIK3CA* mutation was more frequently observed in the LK-TNBC and TCGA-luminal A datasets than in the TNAC and TCGA-TNBC datasets. Furthermore, the *PIK3R1* p.M326I mutation identified from one TNAC patient was dominant in luminal type cases, especially in the luminal A subtype^40^ (Fig. 1B).

### Mutational signatures

**Fig. 2:**
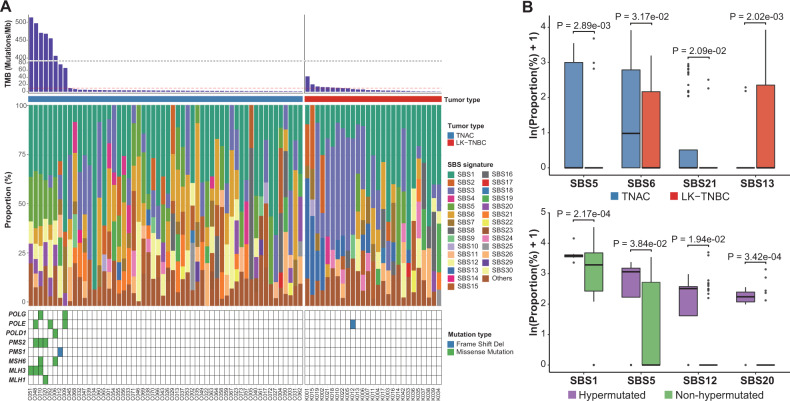
Mutational Signatures in TNAC and LK-TNBC. **A** The proportions of COSMIC mutational signatures (version 2) inferred by deconstructSigs analysis of TNAC and LK-TNBC samples. The top bar plot indicates the TMB of each sample, and a red dotted horizontal line represents the cutoff (10 mutations/Mb) for hypermutation. TNAC and LK-TNBC samples were sorted by TMB. The bottom heatmap represents somatic point mutations in the DNA mismatch repair (MMR)-associated genes. **B** The mutational signatures showing significant differences between TNAC and LK-TNBC (top) and between hypermutated and nonhypermutated samples in TNAC (bottom) (*p* < 0.05). *P* values were calculated by Student’s *t* test. The lower and upper limits of the boxes indicate the 25th and 75th percentiles, respectively, and the horizontal bold line within each box represents the median. Outliers are separately plotted as circles.

Based on COSMIC single base substitution (SBS) signatures, the proportions of 30 SBS signatures were calculated for all TNAC and LK-TNBC samples using the deconstructSigs algorithm (Fig. 2A). We identified 28 mutational signatures in total, and SBS1 was observed in almost all BC samples. In detail, defective DNA mismatch repair (MMR)-related signatures (SBS6 and SBS21) and the SBS5 signature were more enriched in TNAC than in LK-TNBC. In addition, MMR-associated genes were more frequently mutated in TNAC (8/56, 14.29%) than in LK-TNBC (1/27, 3.70%), although the difference was not significant (Fisher’s exact test, *P* = 0.2595). However, an APOBEC activity-associated mutational signature (SBS13) was more prominent in LK-TNBC (Student’s *t* test, *p* < 0.05) (Fig. 2B).

We found eight TNAC and seven LK-TNBC samples with a high tumor mutational burden (TMB) (> 10 mutations per Mb) (Fig. 2A). High-TMB TNAC samples had more than 20 times higher TMB values (mean value: 368.72) than high-TMB LK-TNBC samples (mean value: 17.85). Previously, APOBEC activity (SBS2 and SBS13) and MMR-related mutational signatures (SBS3, SBS6, SBS10, SBS15, and SBS20) were reported to be the most common in high-TMB BC samples^41^. In our TNAC cohort, SBS20 was significantly enriched in hypermutated samples compared to non-hypermutated samples (Student’s *t* test, *p* < 0.05) (Fig. 2B). Interestingly, all MMR gene mutations were identified only in the eight hypermutated TNAC samples. The *MLH3*:p.R1251W variant of C051 was identified by visual inspection with an integrative genomics viewer.

### Somatic copy number alterations

Overall, somatic copy number alterations (SCNAs) in the entire genome were less prevalent in TNAC than in LK-TNBC (Wilcoxon signed-rank test, *p* < 0.05) (Supplementary Fig. 1). However, TNAC had significantly more frequent amplification of 7p and 8q, and deletion of 6q, 9p, and 18 than LK-TNBC, with *q*-values less than 0.05 based on a GISTIC^20^ analysis (Supplementary Table 2). All the TNAC-specific broad events of significant gains and losses were frequently observed in the luminal A subtypes of the TCGA BRCA dataset^5^. In addition, 6q was reported to be frequently deleted in the luminal androgen receptor (LAR) subtype among Burstein TNBC subtypes^26^.

For focal SCNAs, we identified four significant TNAC-specific focal deletions in 1q44, 9p21.3, 14q32.33, and 17p12 and one focal amplification in 5q35.3 (*q*-value < 0.05, Supplementary Table 2). 9p21.3 focal deletion spanning *CDKN2A* and 17p12 deletion overlapping *MAP2K4* are associated with the luminal A subtype of the TCGA breast cancer cohort^5^. Furthermore, 9p21 deletion covering *CDKN2A* was reported to be frequent in the Burstein LAR subtype^26^ and FUSCC LAR subtype^28^ patients. Other focal SCNA information is described in Supplementary Table 2.

### Intrinsic subtype

**Fig. 3:**
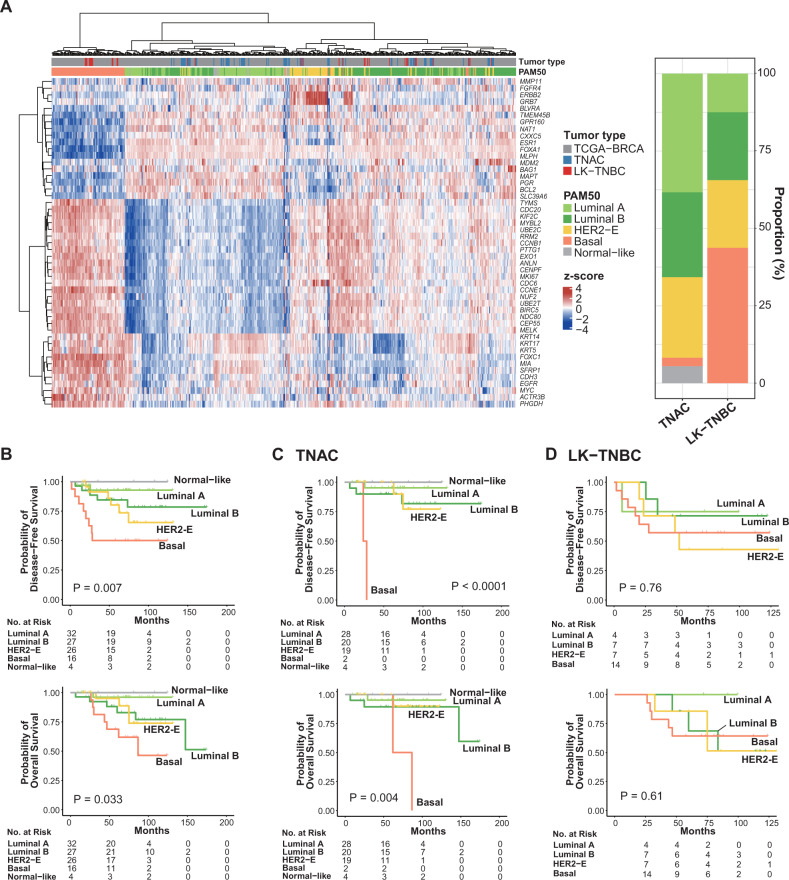
Intrinsic subtyping of TNAC and LK-TNBC. **A** Hierarchical clustering of expression profiles of our cohort and the TCGA BRCA cohort. Rows in the heatmap correspond to PAM50 genes, and columns correspond to individual patients. Gene expression was normalized to Z score; red indicates upregulation, and blue indicates downregulation. The right bar plot shows the proportions of intrinsic subtypes of TNAC and LK-TNBC as determined by PAM50 prediction using ‘genefu’ in the R package (v2.26.0). Differences in disease-free survival (DFS) and overall survival (OS) according to intrinsic subtype of total samples (**B**), TNAC samples (**C**), and LK-TNBC samples (**D**). *P* values were calculated by Kaplan‒Meier survival analysis.

PAM50 prediction was performed using 73 TNACs, 32 LK-TNBCs, and 466 BCs from the TCGA BRCA dataset (Fig. 3A). Of the 73 TNACs, 28 (38.4%) were classified as luminal A, 20 (27.4%) as luminal B, and 19 (26.0%) as HER2-enriched (HER2-E) subtype. Only two (2.7%) TNAC samples were categorized as basal, while four (5.5%) were classified as normal-like. In contrast, in LK-TNBCs, the basal intrinsic subtype was the most dominant subtype (14/32, 43.8%), followed by luminal B (7/32, 21.9%), HER2-E (7/32, 21.9%), and luminal A (4/32, 12.5%) (*p* < 0.001). Intrinsic subtypes showed significantly different prognoses by Kaplan‒Meier survival analysis (Fig. 3B). The basal subtype had the worst survival outcome, whereas the normal-like and luminal A subtypes had better outcomes than the other subtypes, consistent with a previous study^42^ ([5-year DFS of basal, HER2-E, luminal B, luminal A, and normal-like: 50.0%, 80.2%, 84.5%, 92.8%, and 100%, respectively, *P* = 0.007] and [5-year OS of basal, HER2-E, luminal B, luminal A, and normal-like: 68.8%, 95.0%, 82.9%, 96.0%, and 100%, *P* = 0.03]). The intrinsic subtype influenced survival outcome in only TNAC (Fig. 3C, D).

### TNBC subtype

**Fig. 4:**
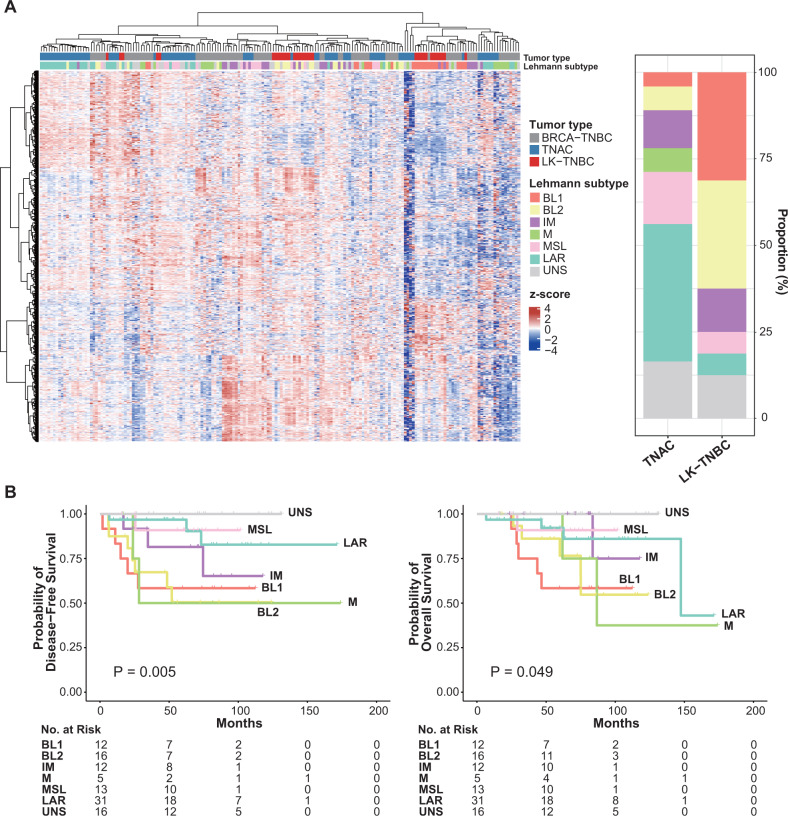
Lehmann Subtyping of TNAC and LK-TNBC. **A** Hierarchical clustering of our cohort and the TCGA TNBC cohort based on expression profiles. Rows in the heatmap correspond to genes distinguishing Lehmann subtypes, and columns correspond to individual patients. Gene expression was normalized to Z score; red indicates upregulation, and blue indicates downregulation. The right bar plot shows the proportion of Lehmann subtypes of TNAC and LK-TNBC, determined by ‘TNBCtype’. **B** Differences in disease-free survival (DFS) and overall survival (OS) according to Lehmann subtype of total samples. *P* values were calculated by Kaplan‒Meier survival analysis.

TNBC subtyping was applied to our TNAC and LK-TNBC cohort and TCGA-TNBC cohort according to Lehmann’s classification^24^. In our TNAC cohort, a luminal androgen receptor (LAR) subtype, which is closely related to breast cancer of the apocrine type^24^, was the most common (29/73, 39.7%), followed by the mesenchymal stem-like (MSL) subtype (11/73, 15.1%), except for unstable (UNS) (Fig. 4A). Only three TNACs (4.1%) were categorized into basal-like (BL) 1, and five (6.8%) were categorized into BL2. BL1 (10/32, 31.3%) and BL2 (10/32, 31.3%) subtypes were frequently observed in LK-TNBC. In LK-TNBC, only two (6.3%) samples were classified as the LAR subtype (*p* < 0.001). TNBC subtype also influenced BC survival outcome (Fig. 4B). The UNS TNBC subtype group did not have any recurrence, and the LAR subtype had a 96.8% 5-year DFS rate. The 5-year DFS rate was 90.9% in MSL, 81.5% in immunomodulatory (IM), 58.3% in BL1, 50.5% in BL2, and 50% in mesenchymal (M) subtype samples (*P* = 0.005). In terms of OS, the UNS group did not experience any death, while the survival rate was 58.3% in BL1, 76.6% in BL2, 100% in IM, 92.2% in LAR, 100% in M, and 90.9% in MSL group (*P* = 0.049).

Burstein^26^ and Fudan University Shanghai Cancer Center (FUSCC)^27^ TNBC subtypes were also evaluated (Supplementary Figs. 2 and 3). The expression levels of a total of 55 genes representing each Burstein subtype were used to predict subtypes of TNAC and LK-TNBC. The cluster with high expression levels of genes related to the Burstein LAR subtype included most TNACs previously assigned to the intrinsic luminal A and Lehmann LAR subtypes. In FUSCC TNBC subtyping, many TNACs (34/73, 46.6%) and TCGA TNBCs with the FUSCC LAR subtype were sorted into the same cluster, whereas only four LK-TNBCs (4/32, 12.5%) were included in the FUSCC LAR subtype.

### Differentially expressed genes in TNAC

Differential gene expression (DGE) analysis between TNAC and LK-TNBC showed that TNAC had 2082 upregulated genes and 162 downregulated genes compared with LK-TNBC (Supplementary Fig. 4, Supplementary Table 3). The level of expression of *CTNNB1*, which was enriched in basal-like breast cancer and associated with a poor prognosis^43^, significantly decreased in TNAC. However, apocrine carcinoma-associated genes such as *ACSM1*, *FABP7*, and *HMGCS2*^44^ showed significant upregulation in TNAC compared to LK-TNBC (Wilcoxon signed-rank test, *p* < 0.05) (Supplementary Fig. 5, Supplementary Table 4). GSEA revealed that metabolic processes of hormones and acids were enriched in TNAC, while epigenetic regulation of gene expression and chromatin organization-related pathways were significantly enriched in LK-TNBC (Supplementary Table 5).

DGE analysis and GSEA of the intrinsic subtypes of TNAC showed that epithelial mesenchymal transition (EMT) and TNF-alpha signaling pathways were upregulated, but E2F targets and mTORC1 signaling pathways were downregulated in luminal A TNAC samples (Supplementary Fig. 6a, Supplementary Table 6). These results were consistent with previous studies reporting that EMT was involved in the pathogenesis of apocrine carcinoma^45^ and that the E2F target gene set was more enriched in the basal subtype than in the luminal or normal-like subtype^46^. Enriched KEGG pathways and GO biological processes are described in Supplementary Fig. 6b, c and Supplementary Table 6.

### Tumor microenvironment of TNAC

Different tumor microenvironments (TMEs) in TNAC and LK-TNBC were identified by the CIBERSORTx algorithm^33^. Macrophages, CD4+ T cells, cancer HER2_sc, and mature luminal cell types were significantly enriched in TNAC compared to LK-TNBC (Wilcoxon signed-rank test, *p* < 0.05, Supplementary Fig. 7). In contrast, cancer cycling and cancer myeloid cell types were more prominent in LK-TNBC than in TNAC (Wilcoxon signed-rank test, *p* < 0.05).

### Gene fusion

*FGFR2-TACC2* fusion, which was discovered as a novel rearrangement in TNAC^39^, was found in one TNAC patient in our cohort. The list of gene fusion events identified from our TNAC and LK-TNBC cohorts is described in Supplementary Table 7.

The frequency of gene fusion events varies significantly depending on tumor type and intrinsic subtype (Supplementary Fig. 8). TNAC showed fewer gene fusion events than LK-TNBC (Wilcoxon signed-rank test, *p* < 0.05). Interestingly, the luminal A subtype had the lowest frequency of gene fusions, and the frequency was significantly increased in the order of luminal B, HER2-E, and basal subtype (Kruskal‒Wallis, *p* < 0.05).

### Survival analysis

**Fig. 5:**
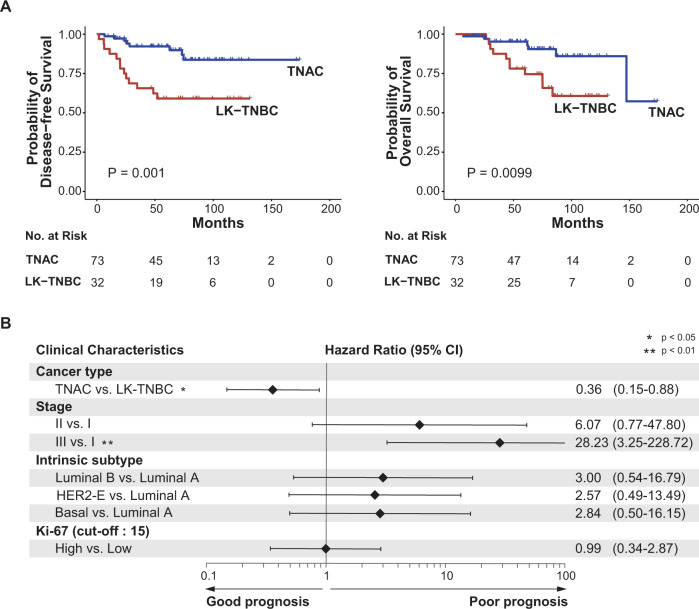
Kaplan‒Meier Survival Analysis and Multivariate Analysis. **A** Kaplan‒Meier survival analysis of DFS and OS in TNAC and LK-TNBC. **B** Cox multivariate analysis of DFS in clinical characteristics. A hazard ratio less than 1 indicates a better prognosis, while a hazard ratio greater than 1 indicates a worse prognosis.

DFS and BC-specific OS were analyzed in both TNAC and LK-TNBC. The median follow-up duration was 71.3 months (interquartile range: 41.2, 90.0). In terms of both DFS and OS, TNAC had superior survival outcomes compared with LK-TNBC. Thus, the five-year DFS rate was 92.2% vs. 59.1% in TNAC vs. LK-TNBC (*P* = 0.001), and the five-year OS rate was 95.3% vs. 74.6% (*P* = 0.0099) (Fig. 5A). Among other clinical characteristics, stage and Ki-67 were associated with BC prognosis (Supplementary Fig. 9a, b). Additional survival analyses were performed on TNAC and LK-TNBC of the same stage. In stage II, TNAC had better DFS than LK-TNBC (Supplementary Fig. 9c).

We performed multivariate analysis of tumor type (TNAC vs. LK-TNBC), stage, Ki-67, and intrinsic subtype. In this analysis, TNAC had better DFS than LK-TNBC. The hazard ratio [HR] of TNAC compared to LK-TNBC was 0.360 with a 95% confidence interval [CI]: 0.148, 0.878 (*P* = 0.02). Stage also significantly affected DFS with an HR of 6.070 for stage II vs. stage I (95% CI: 0.771, 47.801, *P* = 0.08) and an HR of 28.266 for stage III (95% CI: 3.483, 228.719, *P* = 0.002) (Fig. 5B).

## Discussion

In this study, we revealed that TNAC had different genomic characteristics compared to TNBC with low Ki-67. *TP53* and *PIK3CA* mutations were less frequently observed in TNAC than in LK-TNBC, and other mutations were found at different frequencies between TNAC and LK-TNBC. *PIK3R1* mutation was observed only in TNAC. The APOBEC signature was more dominant in LK-TNBC, whereas high TMB was more frequently observed in TNAC and was associated with mutation of MMR-associated genes. Considering intrinsic subtypes, the luminal A subtype was more frequent in TNAC, while the basal subtype was more common in LK-TNBC. DFS and OS were different for TNAC and LK-TNBC. In addition, TNBC subtypes differed between TNAC and LK-TNBC.

In TNBC, *TP53* was the most commonly detected mutation. In the TCGA dataset, ~80% of TNBCs harbored *TP53* mutations, while only 9% harbored *PIK3CA* mutations^5^. Another study suggested that *TP53* mutation was found in 80% of ER-negative BCs, with *PIK3CA* mutation found at a frequency of 20%^47^. In our study, *TP53* mutation was observed in 28.5% of TNACs and 70.4% of LK-TNBCs. *PIK3CA* mutation was observed in 44.4% of LK-TNBCs, similar to the rate in luminal A type samples in the TCGA cohort^5^. *PIK3R1* mutation was found in ~1–3% of BCs regardless of HR and HER2 status^5^. Our study suggested that ~10% of *PIK3R1* mutations were exclusively observed in TNAC.

Ki-67 is one of the most important prognostic biomarkers in TNBC. A previous study suggested that high Ki-67 indicated a poor prognosis compared to low Ki-67 in TNBC^48^. Recent studies reported that TNAC had a lower Ki-67 score and better prognosis than TNBC^3,49^. However, the researchers did not perform multivariate analysis or evaluation of factors affecting prognosis between TNAC or Ki-67 score. Therefore, we analyzed the genetic characteristics and prognosis of TNAC and TNBC while considering Ki-67 expression. We chose LK-TNBC because TNAC generally has a low Ki-67 score. TNBC with low Ki-67 was not frequently observed, and only 25% of TNBCs had a Ki-67 value less than 30%^48^. In our study, 72% and 92% of TNBC and TNAC patients had Ki-67 less than 30%.

The proportions of mutational signatures also differed between TNAC and LK-TNBC. MMR-associated signatures were dominant in TNAC, whereas APOBEC signatures were more prevalent in TNBC. Hypermutation was also frequently observed in TNAC with genetic alteration of MMR-associated genes. In previous study, the MMR signature is rarely detected in BC, but the APOBEC signature is common^50^. The MMR signature of TNAC showed unique genetic characteristics, suggesting this as a surrogate biomarker for the response to ICIs^51^.

Immune checkpoint inhibitor (ICI) treatment combined with chemotherapy has been approved as a standard treatment strategy for TNBC, and an understanding of the TME is important to treat TNBC patients^52–54^. Currently, programmed death-ligand 1 status and tumor-infiltrating lymphocyte (TIL) levels are predictive biomarkers for ICI treatment response in TNBC. However, additional predictive biomarkers for ICI are needed^54,55^. In our TME analysis, the CD4+ T-cell subset and macrophages were enriched in TNAC compared to TNBC. This suggests the potential applicability of ICI treatment for TNAC, as CD4+ T-cell infiltration is associated with the antitumor immune response and the ICI response^56^.

The intrinsic subtype is a traditional biomarker of BC^6^ and has been used as a surrogate biomarker for adjuvant chemotherapy and BC-specific survival^57,58^. Generally, TNBC is categorized into the basal-like subtype, and hormone receptor+HER2- BC is the luminal A subtype^57^. In survival, the basal-like subtype had worse DFS and OS than the luminal A and B subtypes regardless of TNBC according to immunohistochemistry (IHC) results^57^. In our study, TNAC was categorized into luminal A (38.4%), luminal B (26.0%), HER2-E (26.0%), normal-like (5.5%), and basal (2.7%) despite being classified as TNBC by IHC. In contrast, LK-TNBCs were mostly categorized into the basal subtype (43.8%). In terms of Lehmann’s TNBC subtypes, LAR was the most common in TNAC. TNBC was most frequently categorized into BL1 and BL2 subtypes, whereas only 10% of TNACs were classified as BL1 and BL2. These characteristics were associated with DFS and OS. Moreover, multivariate analysis suggested that BC stage and TNAC were associated with DFS. Although intrinsic subtype and TNAC status were strongly related, TNAC remained an important prognostic factor for DFS after statistical adjustment.

Treatment guideline indicates chemotherapy for early TNBC as systemic induction therapy or postoperative chemotherapy^59^. They recommended no systemic therapy for apocrine tumors because of low-risk endocrine nonresponsive histology. However, apocrine carcinomas are generally categorized into TNBC or HER2 + BC according to IHC, and they need to be treated with chemotherapy and/or anti-HER2 treatment^3^. In our study, TNAC, which was categorized into normal-like and luminal A subtypes, had very good DFS and OS compared to that of other intrinsic subtypes. Therefore, we suggest that early-stage TNAC does not need adjuvant chemotherapy if it is the normal-like or luminal A intrinsic type. This would prevent unnecessary chemotherapy, which induces several toxicities, including alopecia, nausea/vomiting, cytopenia and severe infections^60^.

This study has limitations. First, this study was not a prospective clinical trial with intervention; therefore, we could not evaluate specific drug responses according to TNAC and LK-TNBC. In addition, we generated sequencing data using FFPE and FF tissue samples because of the limited availability of TNAC and LK-TNBC samples. To overcome the batch effect between FFPE and FF tissues, we analyzed sequencing data after batch correction.

This is the first attempt to comprehensively assess the genomic characteristics of TNAC. Moreover, this study evaluates the genetic signatures of TNAC and could inform treatment decisions regarding adjuvant chemotherapy in early TNBC. Further prospective clinical trials and parallel translational research are warranted.

In conclusion, TNAC has different genomic characteristics, including intrinsic subtypes, that influence survival outcome. This genetic information may help to inform decisions relating to adjuvant chemotherapy and to predict survival outcome.

## Supplementary information

Supplementary information

Supplementary Tables

## Data Availability

The datasets generated in this study are available in NCBI SRA at PRJNA836553. A data sharing statement provided by the authors is available with the full text of this article at *Experimental & Molecular Medicine*.

## References

[1] Vranic S (2013). Apocrine carcinoma of the breast: a comprehensive review. Histol. Histopathol..

[2] Ogiya A (2010). Apocrine metaplasia of breast cancer: clinicopathological features and predicting response. Breast Cancer.

[3] Tsutsumi Y (2012). Apocrine carcinoma as triple-negative breast cancer: novel definition of apocrine-type carcinoma as estrogen/progesterone receptor-negative and androgen receptor-positive invasive ductal carcinoma. Jpn J. Clin. Oncol..

[4] Rakha EA, Reis-Filho JS, Ellis IO (2008). Basal-like breast cancer: a critical review. J. Clin. Oncol..

[5] Cancer Genome Atlas, N. (2012). Comprehensive molecular portraits of human breast tumours. Nature.

[6] Perou CM (2000). Molecular portraits of human breast tumours. Nature.

[7] Foulkes WD, Smith IE, Reis-Filho JS (2010). Triple-negative breast cancer. N. Engl J. Med..

[8] Kumar P, Aggarwal R (2016). An overview of triple-negative breast cancer. Arch. Gynecol. Obstet..

[9] Cao L, Niu Y (2020). Triple negative breast cancer: special histological types and emerging therapeutic methods. Cancer Biol. Med..

[10] Vranic S (2015). Immunohistochemical and molecular profiling of histologically defined apocrine carcinomas of the breast. Hum. Pathol..

[11] Trapani D (2021). Benefit of adjuvant chemotherapy in patients with special histology subtypes of triple-negative breast cancer: a systematic review. Breast Cancer Res. Treat..

[12] Leon-Ferre RA (2018). Impact of histopathology, tumor-infiltrating lymphocytes, and adjuvant chemotherapy on prognosis of triple-negative breast cancer. Breast Cancer Res. Treat..

[13] Thike AA (2014). Loss of androgen receptor expression predicts early recurrence in triple-negative and basal-like breast cancer. Mod. Pathol..

[14] Li H, Durbin R (2009). Fast and accurate short read alignment with Burrows-Wheeler transform. Bioinformatics.

[15] DePristo MA (2011). A framework for variation discovery and genotyping using next-generation DNA sequencing data. Nat. Genet..

[16] Cibulskis K (2013). Sensitive detection of somatic point mutations in impure and heterogeneous cancer samples. Nat. Biotechnol..

[17] Fortunato, A. et al. A new method to accurately identify single nucleotide variants using small FFPE breast samples. *Brief Bioinform.* **22**, 10.1093/bib/bbab221 (2021). 10.1093/bib/bbab221 PMC8574974 34117742

[18] Martincorena I (2018). Universal patterns of selection in cancer and somatic tissues. Cell.

[19] Rosenthal R, McGranahan N, Herrero J, Taylor BS, Swanton C (2016). DeconstructSigs: delineating mutational processes in single tumors distinguishes DNA repair deficiencies and patterns of carcinoma evolution. Genome Biol..

[20] Mermel CH (2011). GISTIC2.0 facilitates sensitive and confident localization of the targets of focal somatic copy-number alteration in human cancers. Genome Biol..

[21] Zhang Y, Parmigiani G, Johnson WE (2020). ComBat-seq: batch effect adjustment for RNA-seq count data. NAR Genom. Bioinform..

[22] Johnson WE, Li C, Rabinovic A (2007). Adjusting batch effects in microarray expression data using empirical Bayes methods. Biostatistics.

[23] Gendoo DM (2016). Genefu: an R/Bioconductor package for computation of gene expression-based signatures in breast cancer. Bioinformatics.

[24] Lehmann BD (2011). Identification of human triple-negative breast cancer subtypes and preclinical models for selection of targeted therapies. J. Clin. Invest..

[25] Chen X (2012). TNBCtype: a subtyping tool for triple-negative breast cancer. Cancer Inform..

[26] Burstein MD (2015). Comprehensive genomic analysis identifies novel subtypes and targets of triple-negative breast cancer. Clin. Cancer Res..

[27] Liu YR (2016). Comprehensive transcriptome analysis identifies novel molecular subtypes and subtype-specific RNAs of triple-negative breast cancer. Breast Cancer Res..

[28] Jiang YZ (2019). Genomic and transcriptomic landscape of triple-negative breast cancers: subtypes and treatment strategies. Cancer Cell.

[29] Subramanian A (2005). Gene set enrichment analysis: a knowledge-based approach for interpreting genome-wide expression profiles. Proc. Natl Acad. Sci. USA.

[30] Tarazona S (2015). Data quality aware analysis of differential expression in RNA-seq with NOISeq R/Bioc package. Nucleic Acids Res..

[31] Chen EY (2013). Enrichr: interactive and collaborative HTML5 gene list enrichment analysis tool. BMC Bioinformatics.

[32] Wu SZ (2021). A single-cell and spatially resolved atlas of human breast cancers. Nat. Genet..

[33] Newman AM (2019). Determining cell type abundance and expression from bulk tissues with digital cytometry. Nat. Biotechnol..

[34] Wang X, Park J, Susztak K, Zhang NR, Li M (2019). Bulk tissue cell type deconvolution with multi-subject single-cell expression reference. Nat. Commun..

[35] Jew B (2020). Accurate estimation of cell composition in bulk expression through robust integration of single-cell information. Nat. Commun..

[36] Uhrig S (2021). Accurate and efficient detection of gene fusions from RNA sequencing data. Genome Res..

[37] Jeon S (2020). Korean Genome Project: 1094 Korean personal genomes with clinical information. Sci. Adv..

[38] Weisman PS (2016). Genetic alterations of triple negative breast cancer by targeted next-generation sequencing and correlation with tumor morphology. Mod. Pathol..

[39] Sun X (2020). Invasive apocrine carcinoma of the breast: clinicopathologic features and comprehensive genomic profiling of 18 pure triple-negative apocrine carcinomas. Mod. Pathol..

[40] Chen L (2018). Characterization of PIK3CA and PIK3R1 somatic mutations in Chinese breast cancer patients. Nat. Commun..

[41] Barroso-Sousa R (2020). Prevalence and mutational determinants of high tumor mutation burden in breast cancer. Ann. Oncol..

[42] Nielsen TO (2010). A comparison of PAM50 intrinsic subtyping with immunohistochemistry and clinical prognostic factors in tamoxifen-treated estrogen receptor-positive breast cancer. Clin Cancer Res..

[43] Khramtsov AI (2010). Wnt/beta-catenin pathway activation is enriched in basal-like breast cancers and predicts poor outcome. Am. J. Pathol..

[44] Gromov P (2014). FABP7 and HMGCS2 are novel protein markers for apocrine differentiation categorizing apocrine carcinoma of the breast. PLoS One.

[45] Vranic, S. & Gatalica, Z. An update on the molecular and clinical characteristics of apocrine carcinoma of the breast. *Clin. Breast Cancer*, 10.1016/j.clbc.2021.12.009 (2021). 10.1016/j.clbc.2021.12.009 35027319

[46] Oshi, M. et al. The E2F pathway score as a predictive biomarker of response to neoadjuvant therapy in ER+/HER2- breast cancer. *Cells* **9**, 10.3390/cells9071643 (2020). 10.3390/cells9071643 PMC7407968 32650578

[47] Nik-Zainal S (2016). Landscape of somatic mutations in 560 breast cancer whole-genome sequences. Nature.

[48] Zhu X (2020). The prognostic and predictive potential of Ki-67 in triple-negative breast cancer. Sci. Rep..

[49] Hu, T. et al. Triple-negative apocrine breast carcinoma has better prognosis despite poor response to neoadjuvant chemotherapy. *J. Clin. Med.* **11**, 10.3390/jcm11061607 (2022). 10.3390/jcm11061607 PMC8949126 35329934

[50] Alexandrov LB (2020). The repertoire of mutational signatures in human cancer. Nature.

[51] Andre T (2020). Pembrolizumab in microsatellite-instability-high advanced colorectal cancer. N. Engl J. Med..

[52] Binnewies M (2018). Understanding the tumor immune microenvironment (TIME) for effective therapy. Nat. Med..

[53] Shah M (2022). FDA approval summary: pembrolizumab for neoadjuvant and adjuvant treatment of patients with high-risk early-stage triple-negative breast cancer. Clin. Cancer Res..

[54] Cortes J (2022). Pembrolizumab plus chemotherapy in advanced triple-negative breast cancer. N. Engl J. Med..

[55] Loi S (2021). The journey of tumor-infiltrating lymphocytes as a biomarker in breast cancer: clinical utility in an era of checkpoint inhibition. Ann. Oncol..

[56] Tay RE, Richardson EK, Toh HC (2021). Revisiting the role of CD4(+) T cells in cancer immunotherapy-new insights into old paradigms. Cancer Gene Ther..

[57] Carey LA (2006). Race, breast cancer subtypes, and survival in the Carolina Breast Cancer Study. JAMA.

[58] Cardoso F (2016). 70-gene signature as an aid to treatment decisions in early-stage breast cancer. N. Engl J. Med..

[59] Cardoso F (2019). Early breast cancer: ESMO Clinical Practice Guidelines for diagnosis, treatment and follow-updagger. Ann. Oncol..

[60] Sparano JA (2008). Weekly paclitaxel in the adjuvant treatment of breast cancer. N. Engl J. Med..

