# Supplementary material for: Genomic characteristics of triple negative apocrine carcinoma: a comparison to triple negative breast cancer
Source: Exp Mol Med. 2023 Jul 3;55(7):1451–61. doi: 10.1038/s12276-023-01030-z (PMC10394068; doi:10.1038/s12276-023-01030-z)
Supplement: Supplementary file 1 — Supplementary information [file 12276_2023_1030_MOESM1_ESM.pdf]

Genomic Characteristics of Triple Negative Apocrine Carcinoma: A Comparison to Triple Negative Breast Cancer

Ji-Yeon Kim, Sabin Park, Eun Yoon Cho, Jeong Eon Lee, Hae Hyun Jung, Byung Joo Chae, Seok Won Kim, Seok Jin Nam, Soo Youn Cho, Yeon Hee Park, Jin Seok Ahn, Semin Lee, Young-Hyuck Im

This file includes:

Supplementary Figure 1-9

Supplementary Figure Legends

Supplementary Table 1-7 : We provide supplementary tables as excel file.

Supplementary Figure 1.

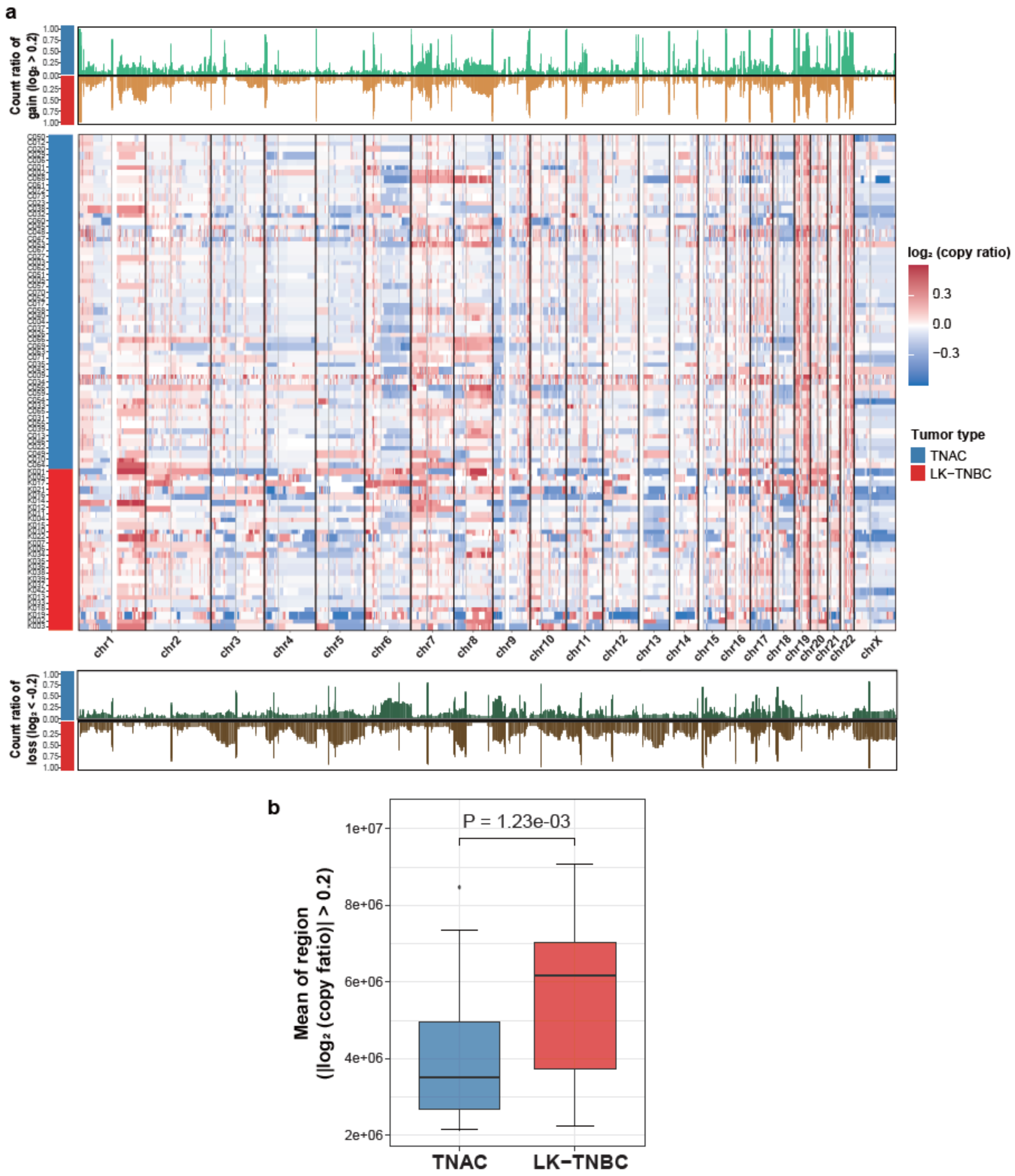

Supplementary Figure 2.

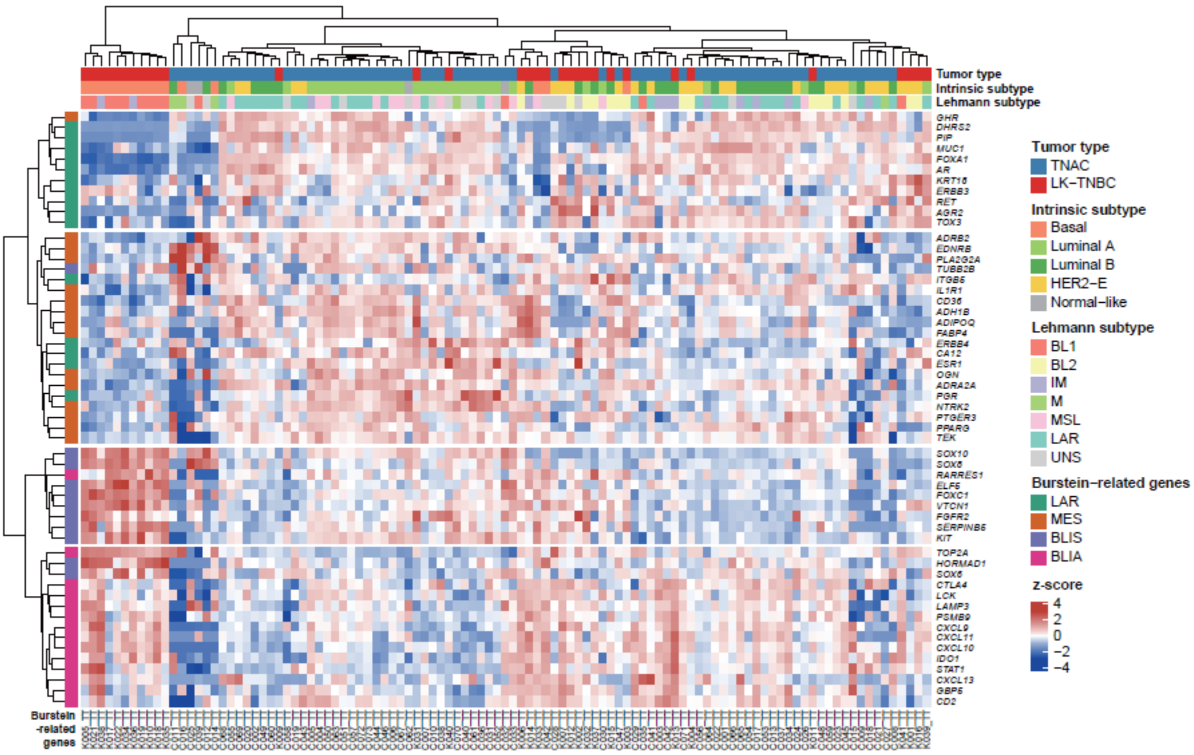

Supplementary Figure 3.

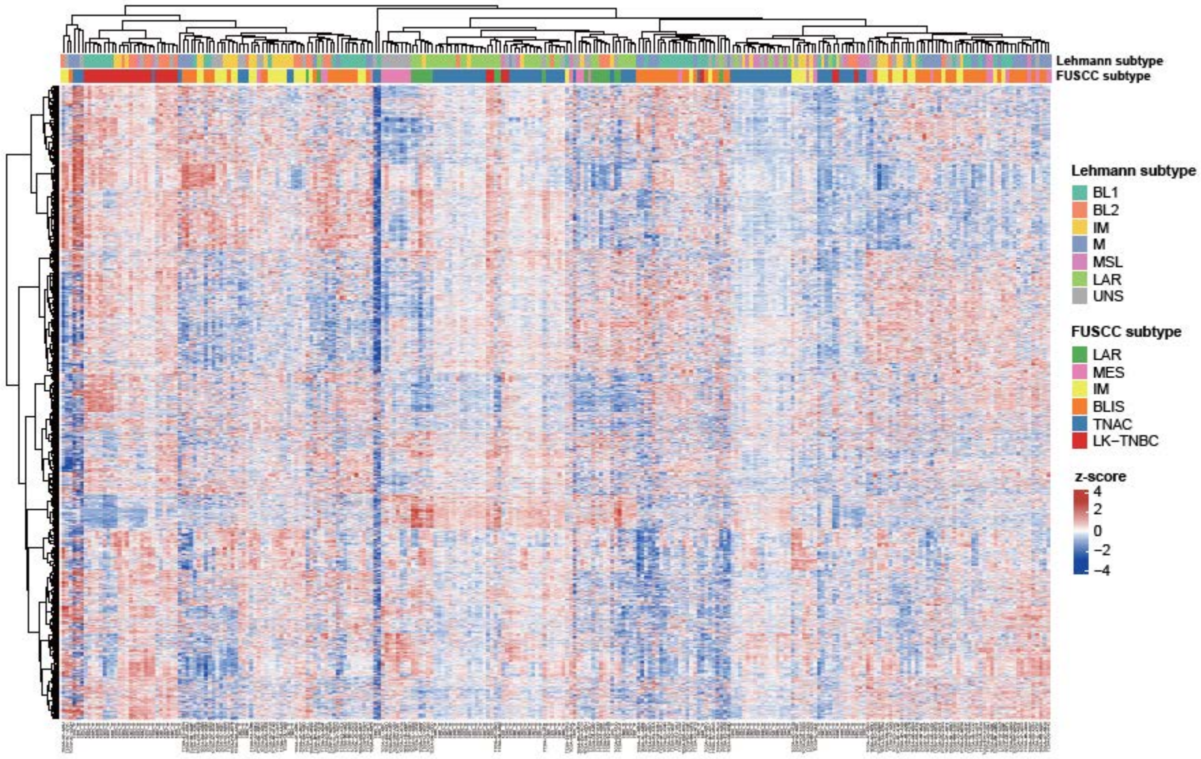

Supplementary Figure 4.

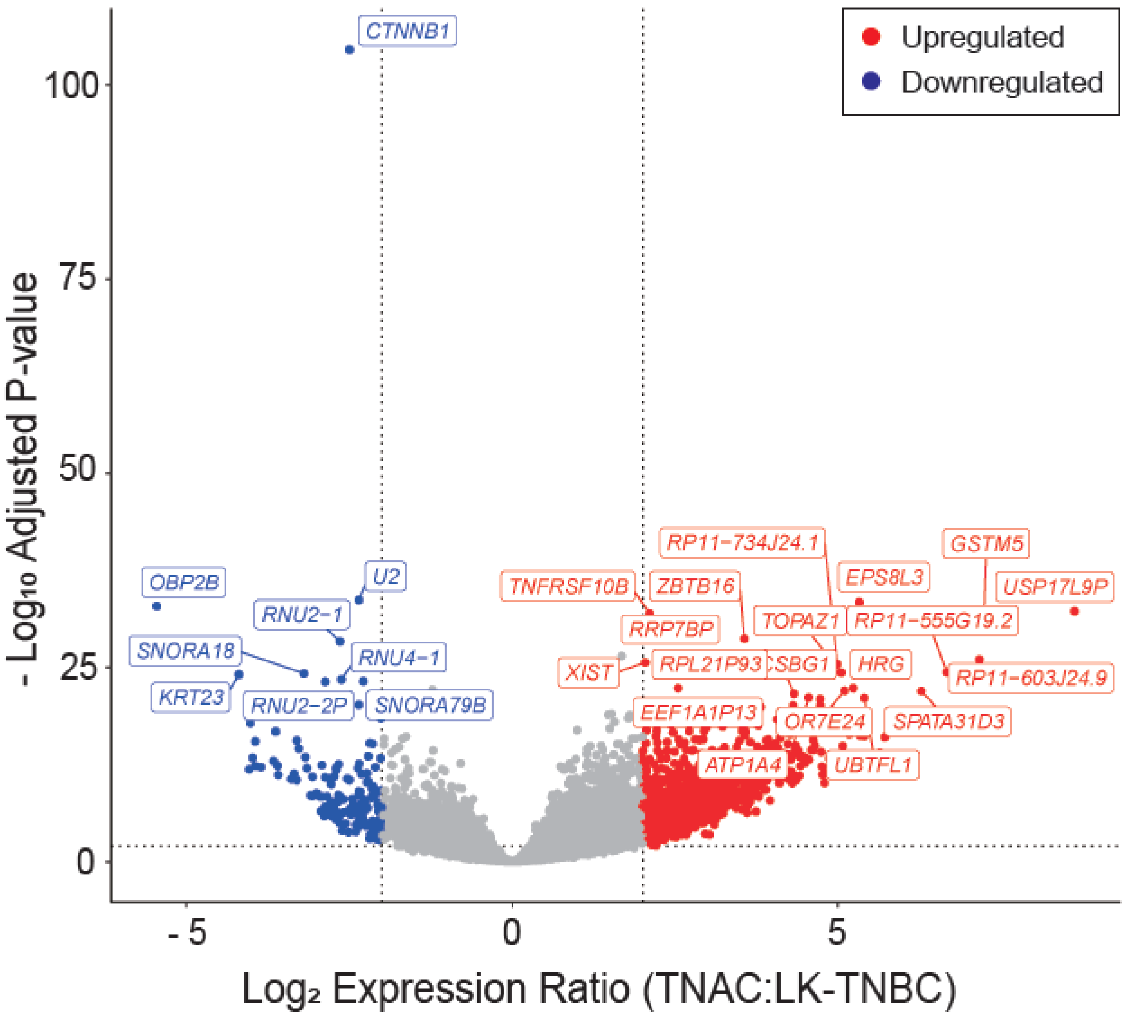

Supplementary Figure 5.

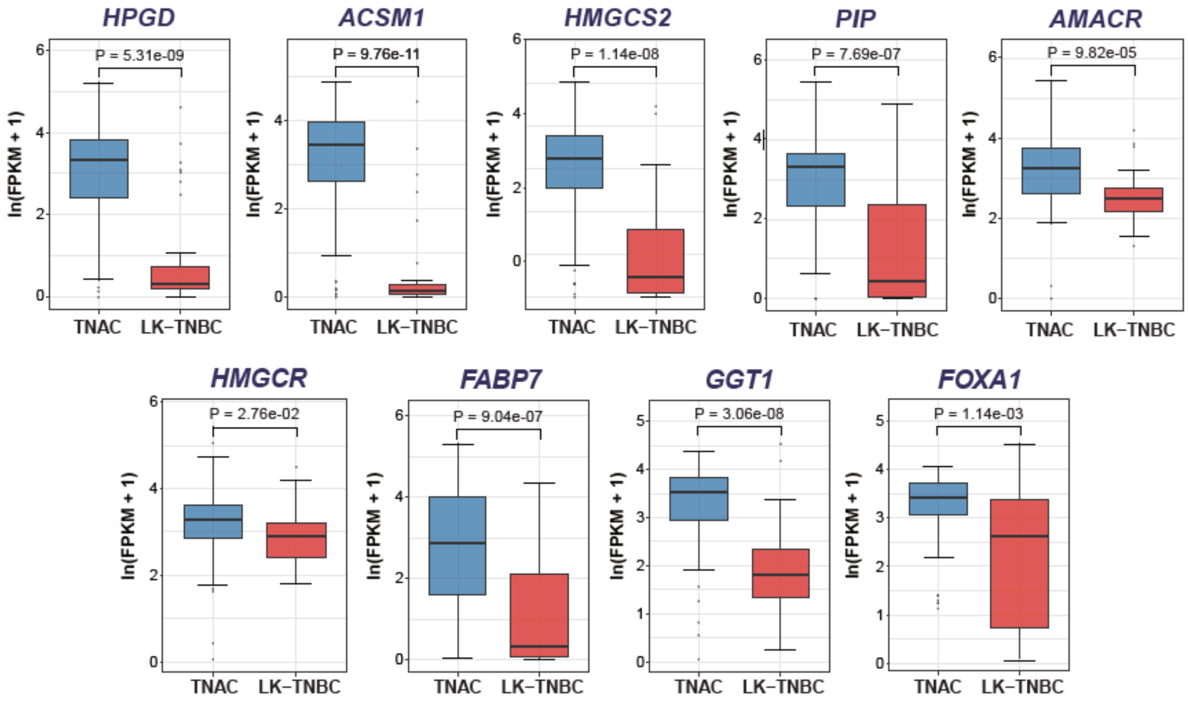

Supplementary Figure 6.

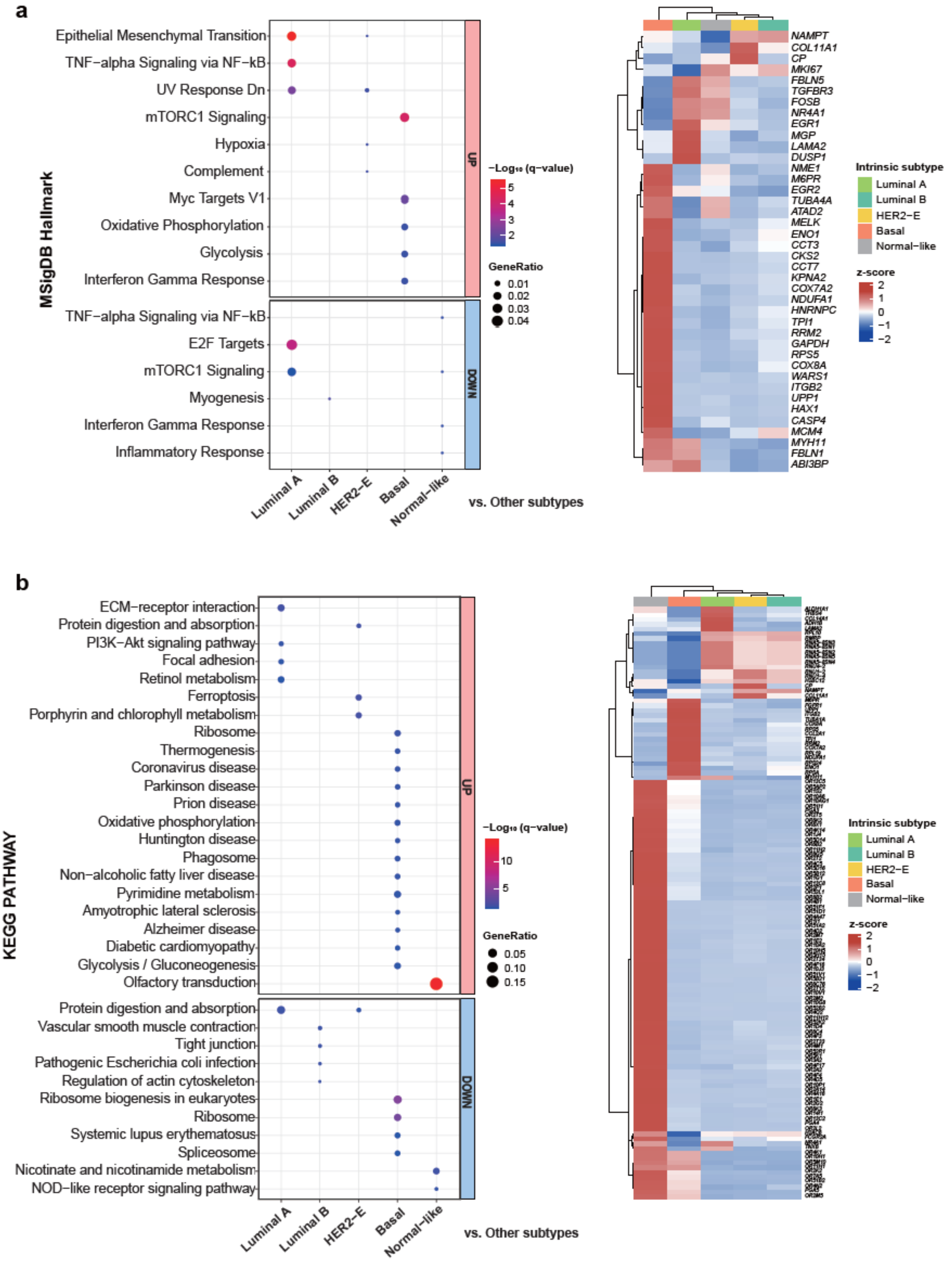

c

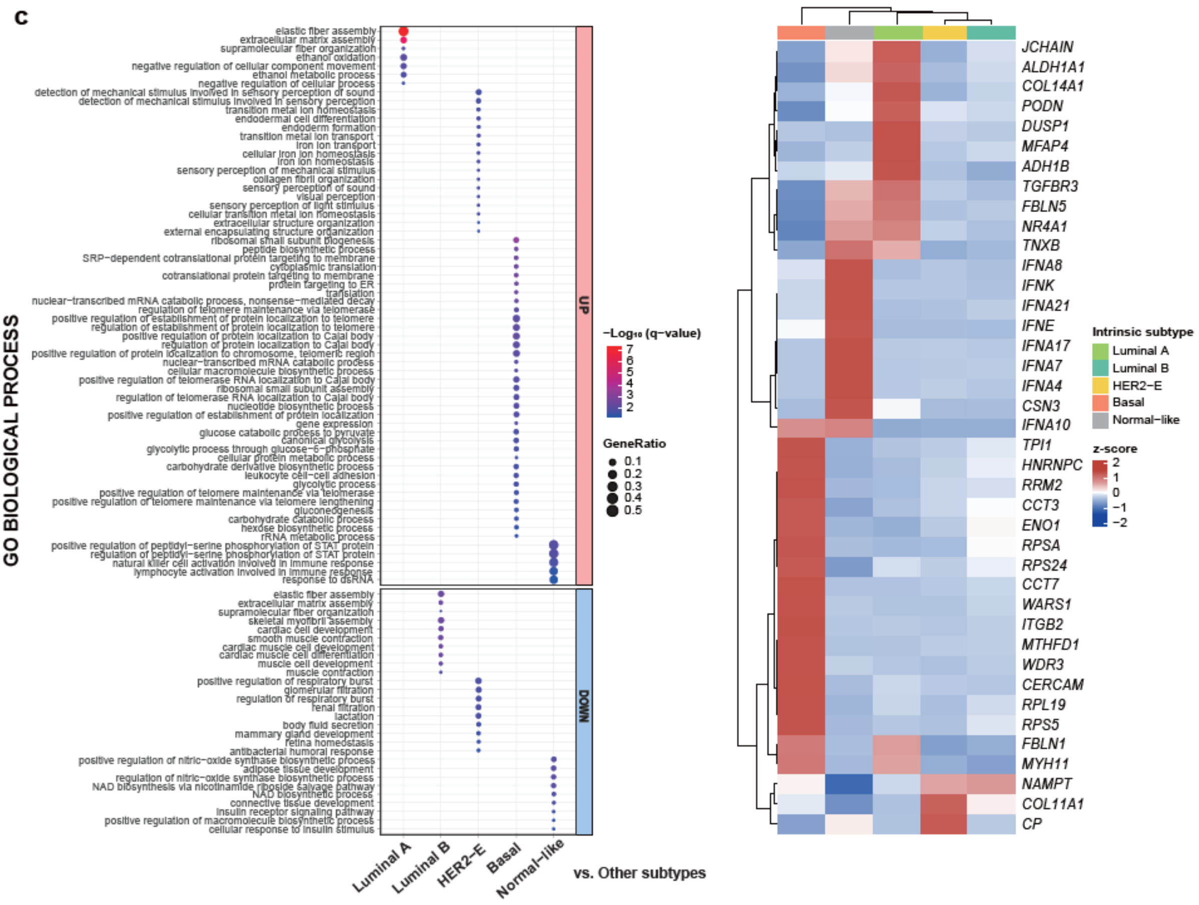

GO BIOLOGICAL PROCESS
elastic fiber assembly
extracellular matrix assembly
supramolecular fiber organization
ethanol oxidation
negative regulation of cellular component movement
ethanol metabolic process
negative regulation of cellular process
detection of mechanical stimulus involved in sensory perception of sound
detection of mechanical stimulus involved in sensory perception
transition metal ion homeostasis
endodermal cell differentiation
endoderm formation
transition metal ion transport
iron ion transport
cellular iron ion homeostasis
iron ion homeostasis
sensory perception of mechanical stimulus
collagen fibril organization
sensory perception of sound
visual perception
sensory perception of light stimulus
cellular transition metal ion homeostasis
extracellular structure organization
external encapsulating structure organization
ribosomal small subunit biogenesis
peptide biosynthetic process
SRP−dependent cotranslational protein targeting to membrane
cytoplasmic translation
cotranslational protein targeting to membrane
protein targeting to ER
translation
nuclear−transcribed mRNA catabolic process, nonsense−mediated decay
regulation of telomere maintenance via telomerase
positive regulation of establishment of protein localization to telomere
regulation of establishment of protein localization to telomere
positive regulation of protein localization to Cajal body
regulation of protein localization to Cajal body
positive regulation of protein localization to chromosome, telomeric region
nuclear−transcribed mRNA catabolic process
cellular macromolecule biosynthetic process
positive regulation of telomerase RNA localization to Cajal body
ribosomal small subunit assembly
regulation of telomerase RNA localization to Cajal body
nucleotide biosynthetic process
positive regulation of establishment of protein localization
gene expression
glucose catabolic process to pyruvate
canonical glycolysis
glycolytic process through glucose−6−phosphate
cellular protein metabolic process
carbohydrate derivative biosynthetic process
leukocyte cell−cell adhesion
glycolytic process
positive regulation of telomere maintenance via telomerase
positive regulation of telomere maintenance via telomere lengthening
gluconeogenesis
carbohydrate catabolic process
hexose biosynthetic process
rRNA metabolic process
positive regulation of peptidyl−serine phosphorylation of STAT protein
regulation of peptidyl−serine phosphorylation of STAT protein
natural killer cell activation involved in immune response
lymphocyte activation involved in immune response
response to dsRNA
UP
elastic fiber assembly
extracellular matrix assembly
supramolecular fiber organization
skeletal myofibril assembly
cardiac cell development
smooth muscle contraction
cardiac muscle cell development
cardiac muscle cell differentiation
muscle cell development
muscle contraction
positive regulation of respiratory burst
glomerular filtration
regulation of respiratory burst
renal filtration
lactation
body fluid secretion
mammary gland development
retina homeostasis
antibacterial humoral response
positive regulation of nitric−oxide synthase biosynthetic process
adipose tissue development
regulation of nitric−oxide synthase biosynthetic process
NAD biosynthesis via nicotinamide riboside salvage pathway
NAD biosynthetic process
connective tissue development
insulin receptor signaling pathway
positive regulation of macromolecule biosynthetic process
cellular response to insulin stimulus
DOWN
Luminal A
Luminal B
HER2−E
Basal
Normal−like
vs. Other subtypes
−Log10 (q−value)
7
6
5
4
3
2
GeneRatio
0.1
0.2
0.3
0.4
0.5
JCHAIN
ALDH1A1
COL14A1
PODN
DUSP1
MFAP4
ADH1B
TGFBR3
FBLN5
NR4A1
TNXB
IFNA8
IFNK
IFNA21
IFNE
IFNA17
IFNA7
IFNA4
CSN3
IFNA10
TPI1
HNRNPC
RRM2
CCT3
ENO1
RPSA
RPS24
CCT7
WARS1
ITGB2
MTHFD1
WDR3
CERCAM
RPL19
RPS5
FBLN1
MYH11
NAMPT
COL11A1
CP
Intrinsic subtype
Luminal A
Luminal B
HER2−E
Basal
Normal−like
z-score
2
1
0
−1
−2

Supplementary Figure 7.

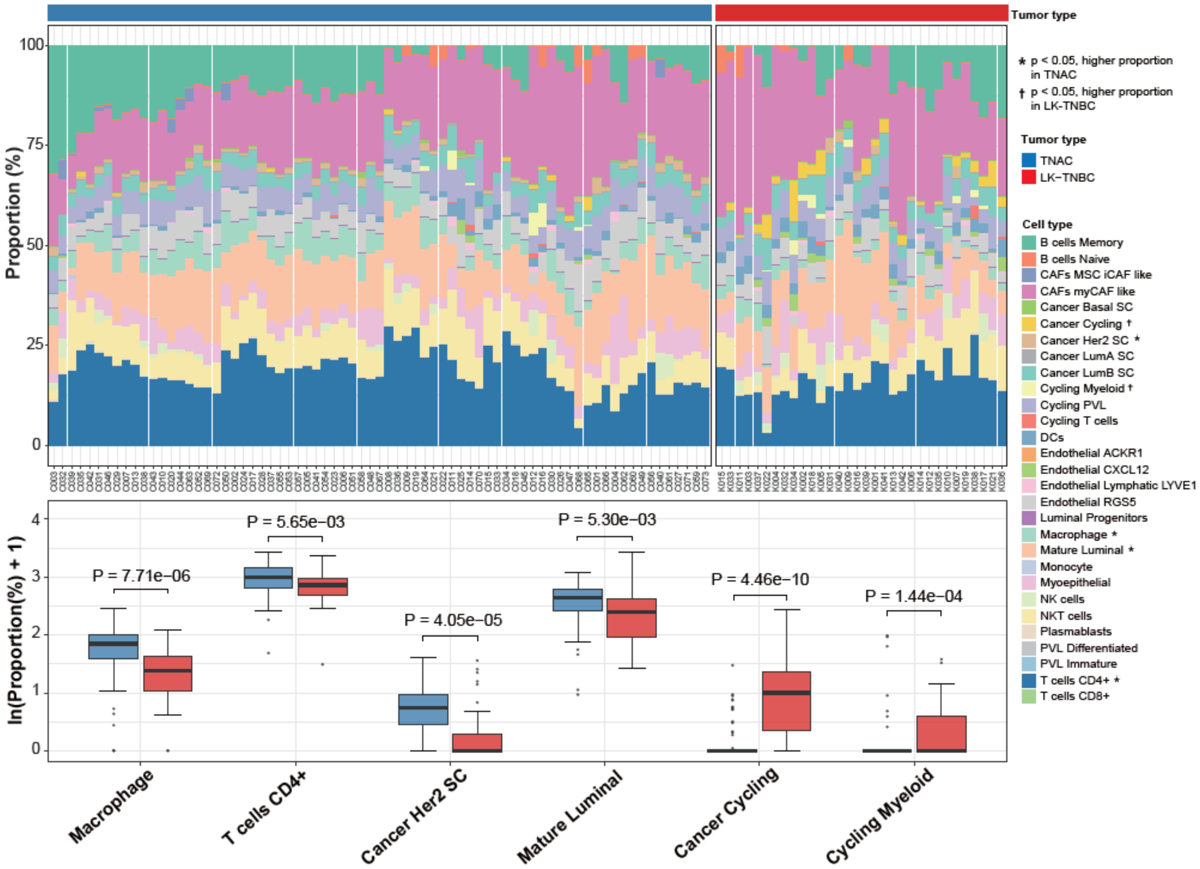

Supplementary Figure 8.

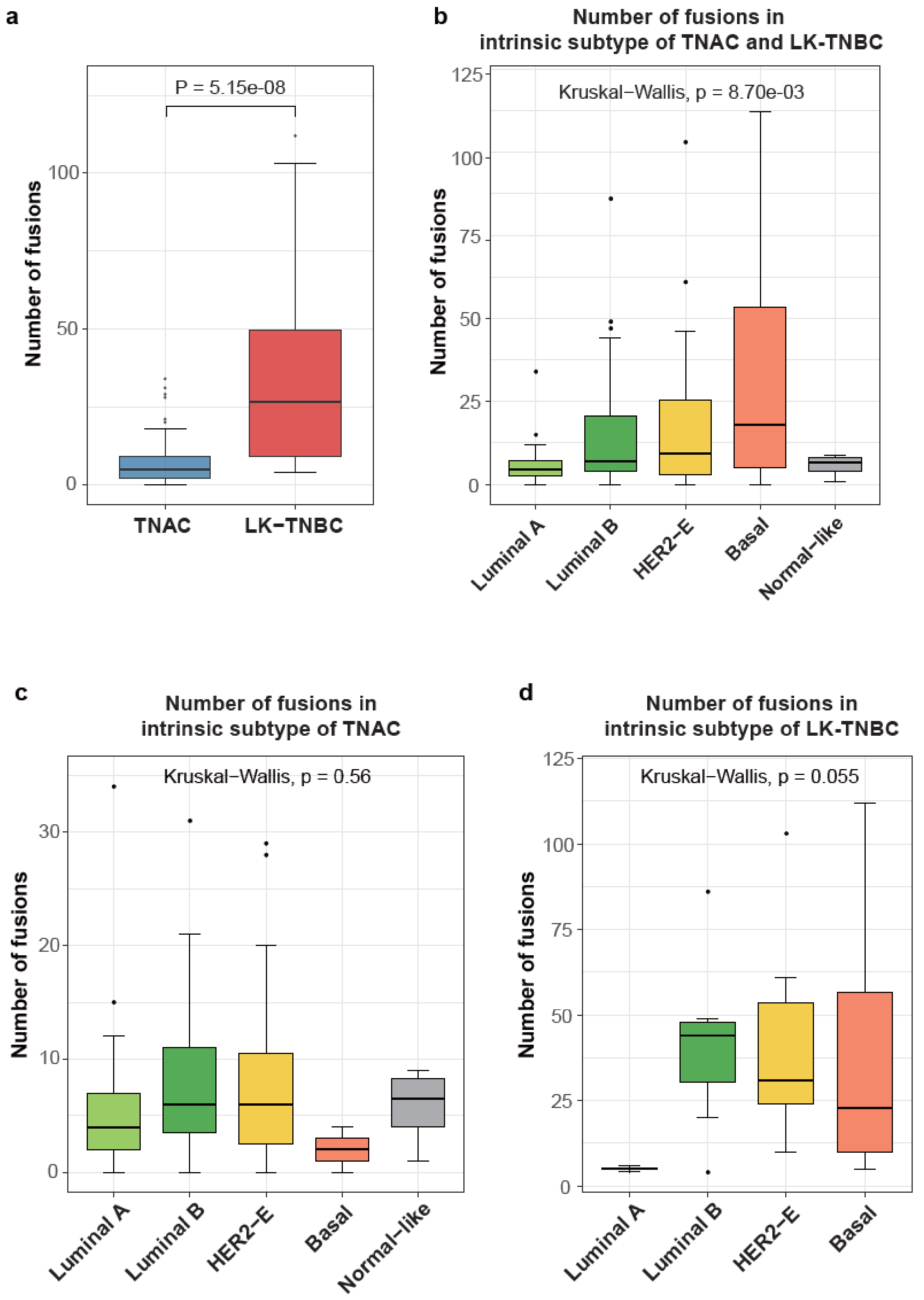

Supplementary Figure 9.

a

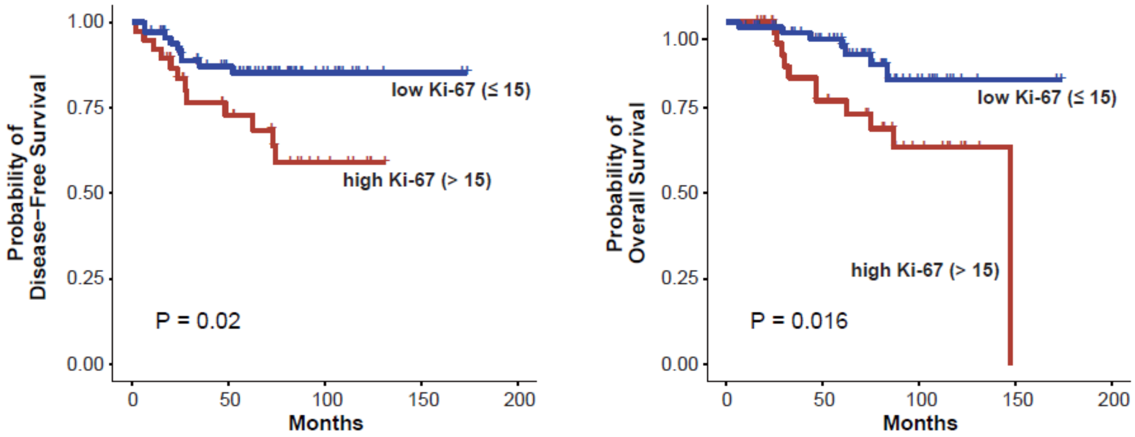

No. at Risk

	0	50	100	150	200
high Ki-67 (> 15)	38	19	7	0	0
low Ki-67 (≤ 15)	67	45	12	2	0

No. at Risk

	0	50	100	150	200
high Ki-67 (> 15)	38	22	9	0	0
low Ki-67 (≤ 15)	67	50	12	2	0

b

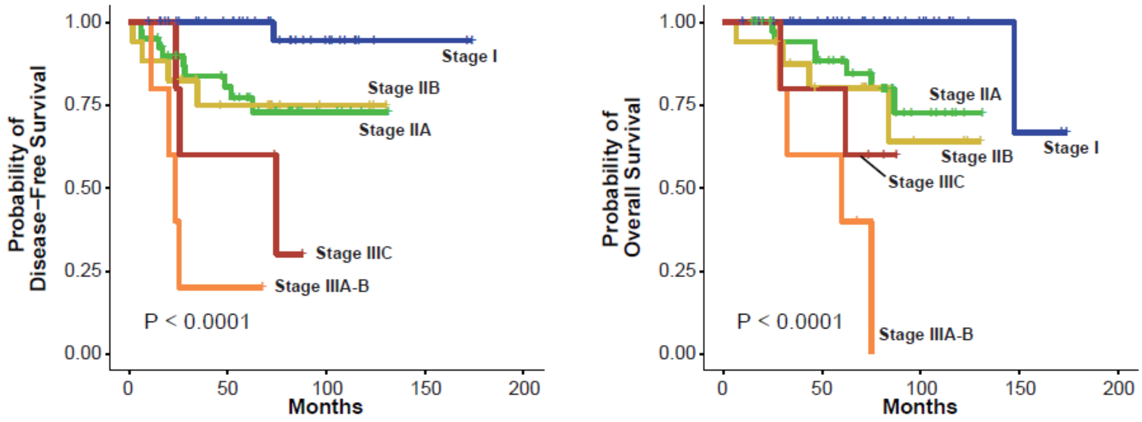

No. at Risk

	0	50	100	150	200
Stage I	38	27	10	2	0
Stage IIA	40	24	6	0	0
Stage IIB	17	9	3	0	0
Stage IIIA-B	5	1	0	0	0
Stage IIIC	5	3	0	0	0

No. at Risk

	0	50	100	150	200
Stage I	38	27	11	2	0
Stage IIA	40	28	7	0	0
Stage IIB	17	10	3	0	0
Stage IIIA-B	5	3	0	0	0
Stage IIIC	5	4	0	0	0

c

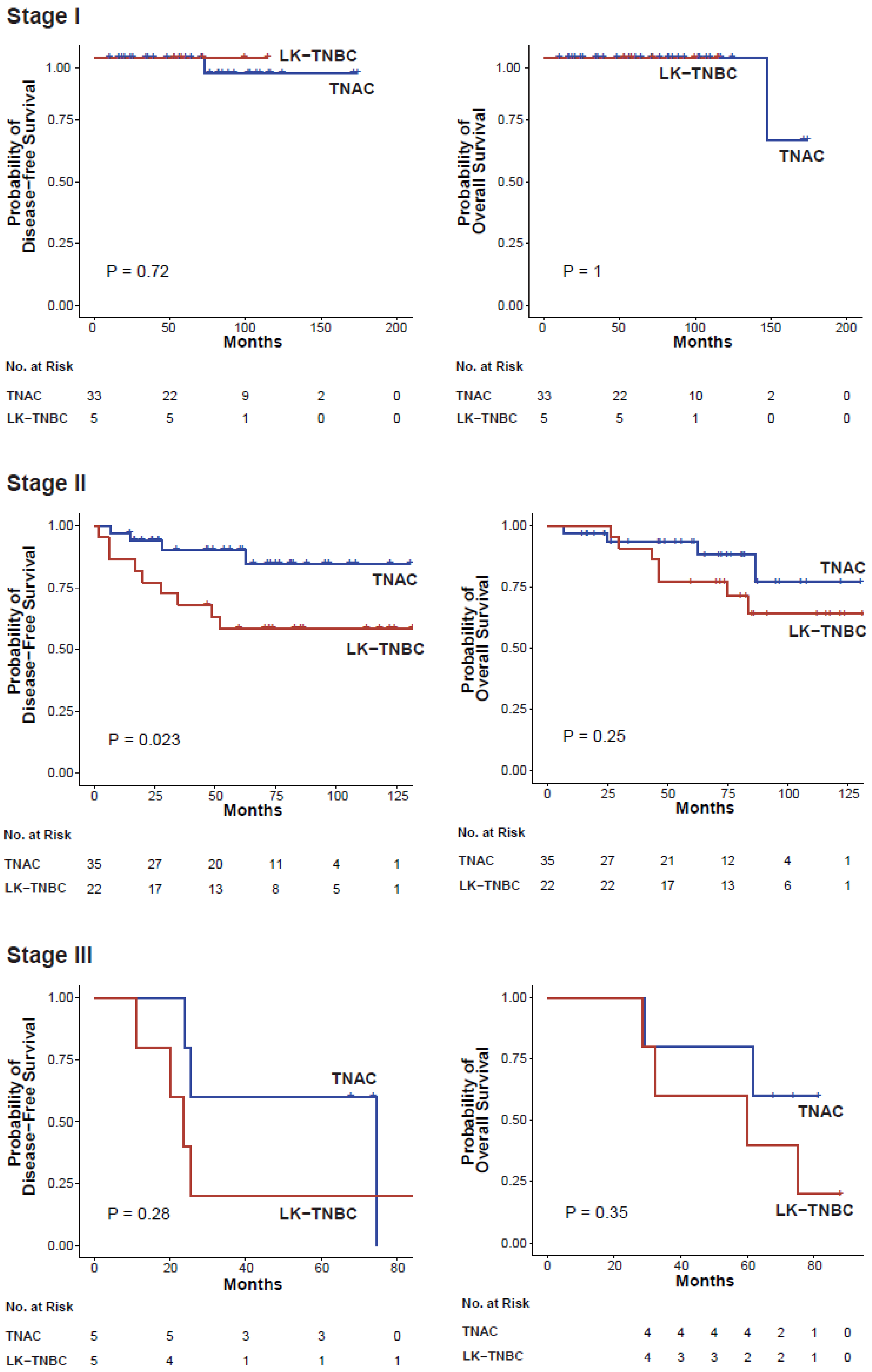
Stage I
Probability of Disease-free Survival
LK-TNBC
TNAC
P = 0.72
Months
No. at Risk
TNAC 33 22 9 2 0
LK-TNBC 5 5 1 0 0
Probability of Overall Survival
LK-TNBC
TNAC
P = 1
Months
No. at Risk
TNAC 33 22 10 2 0
LK-TNBC 5 5 1 0 0
Stage II
Probability of Disease-Free Survival
TNAC
LK-TNBC
P = 0.023
Months
No. at Risk
TNAC 35 27 20 11 4 1
LK-TNBC 22 17 13 8 5 1
Probability of Overall Survival
TNAC
LK-TNBC
P = 0.25
Months
No. at Risk
TNAC 35 27 21 12 4 1
LK-TNBC 22 22 17 13 6 1
Stage III
Probability of Disease-Free Survival
TNAC
LK-TNBC
P = 0.28
Months
No. at Risk
TNAC 5 5 3 3 0
LK-TNBC 5 4 1 1 1
Probability of Overall Survival
TNAC
LK-TNBC
P = 0.35
Months
No. at Risk
TNAC 4 4 4 4 2 1 0
LK-TNBC 4 3 3 2 2 1 0

Supplementary Figure Legends

Supplementary Fig. 1. Somatic copy number alteration (SCNA) profiles of TNAC and LK-TNBC.

(a) SCNA profiles of TNAC and LK-TNBC. In color scale of the heatmap, red indicates copy gain, and blue indicates copy loss. The top and bottom barplots show the frequency of samples which satisfy the cutoff of gain and loss for each chromosome position; $\log_2$ (copy ratio) higher than 0.2 was counted as copy gain, and lower than -0.2 was counted as copy loss.

(b) Difference of the SCNA region size ($|\log_2$ (copy ratio)$| > 0.2$) between TNAC and LK-TNBC. P value was calculated by Wilcoxon signed-rank test. The lower and upper limits of boxes indicate the 25th and 75th percentiles, and the horizontal bold line within each box represents the median. Outliers are separately plotted as circles.

Supplementary Fig. 2. Hierarchical clustering of TNAC and LK-TNBC based on the 55 Burstein subtype genes.

Burstein subtyping was performed by hierarchical clustering of TNAC and LK-TNBC samples using 55 genes significantly overexpressed in each Burstein subtype. Rows in the heatmap correspond to genes, and columns correspond to individual patients. The results of intrinsic and Lehmann subtype prediction were annotated. Gene expression was normalized to Z-score; red indicates up-regulation, and blue indicates down-regulation.

Supplementary Fig. 3. Hierarchical clustering of TNAC, LK-TNBC and TCGA samples based on variable genes in FUSCC classification.

FUSCC classification of TNAC and LK-TNBC was performed by co-clustering analysis of our cohort and TCGA TNBC cohort using the top 2,000 most variable genes in TCGA TNBC patients. Rows in the heatmap correspond to genes, and columns correspond to individual patients. The predicted Lehmann subtypes were annotated. Gene expression was normalized to Z-score; red indicates up-regulation, and blue indicates down-regulation.

Supplementary Fig. 4. Differential gene expression analysis in TNAC vs. LK-TNBC.

A volcano plot shows differences in gene expression between TNAC and LK-TNBC. The x axis indicates the $\log_2$ expression ratio (TNAC:LK-TNBC); greater than 2 indicates genes with higher expression in TNAC relative to LK-TNBC, and less than -2 indicates genes with lower expression in TNAC relative to LK-TNBC. The y axis shows the $-\log_{10}$ adjusted P value, with greater than 2 (adjusted P value < 0.01) representing significant difference between two groups. Grey points indicate genes which have no significant difference. Genes with $|\log_2$ expression ratio (TNAC:LK-TNBC)$| > 2$

and adjusted P value < 1xe^{-18} were labelled in volcano plot.

Supplementary Fig. 5. Expression of apocrine carcinoma-associated genes in TNAC vs. LK-TNBC.

The previously reported apocrine-associated genes showing significant difference between TNAC and LK-TNBC ($p < 0.05$). P values were calculated by Wilcoxon signed-rank test. The lower and upper limits of boxes indicate the 25th and 75th percentiles, and the horizontal bold line within each box represents the median. Outliers are separately plotted as circles.

Supplementary Fig. 6. Gene set enrichment test in intrinsic subtypes of TNAC.

Significantly enriched pathways for up-regulated and down-regulated DEGs among intrinsic subtypes of TNAC according to pathway databases ($q < 0.05$). The color scale of dot plot indicates significance of pathway enrichment, and the size of circles indicates the proportion of genes in each pathway category database that overlap with DEGs. Hierarchical clustering based on the mean expression of pathway related gene in each intrinsic subtype of TNAC was performed. Gene expression was normalized to Z-score; red indicates up-regulation, and blue indicates down-regulation.

(a) Significantly enriched pathways in each intrinsic subtype of TNAC based on MSigDB Hallmark.

(b) Significantly enriched pathways in each intrinsic subtype of TNAC based on KEGG Pathway.

(c) Significantly enriched pathways in each intrinsic subtype of TNAC based on Gene Ontology Biological Process.

Supplementary Fig. 7. Tumor microenvironment (TME) in TNAC and LK-TNBC.

The top barplot shows the proportion of 29 cell types of each TNAC and LK-TNBC patient. Samples were sorted by the result of hierarchical clustering based on cell type proportion profiles. The bottom boxplots show cell types with significantly different proportions between TNAC and LK-TNBC ($p < 0.05$). P values were calculated by Wilcoxon signed-rank test. The lower and upper limits of boxes indicate the 25th and 75th percentiles, and the horizontal bold line within each box represents the median. Outliers are separately plotted as circles.

Supplementary Fig. 8. The number of gene fusions.

(a) Difference of the number of gene fusion events between TNAC and LK-TNBC. P value was calculated by Wilcoxon signed-rank test.

(b) Difference of the number of gene fusion events between intrinsic subtypes of our cohort (TNAC and LK-TNBC). P value was calculated by Kruskal-Wallis test.

(c) Difference of the number of gene fusion events between intrinsic subtypes of TNAC. P value was calculated by Kruskal-Wallis test.

(d) Difference of the number of gene fusion events between intrinsic subtypes of LK-TNBC. P value was calculated by Kruskal-Wallis test.

Supplementary Fig. 9. Survival analysis in Ki-67 and stage.

(a) Kaplan-Meier survival analysis of DFS and OS in Ki-67. High and low Ki-67 were divided based on 15.

(b) Kaplan-Meier survival analysis of DFS and OS in stage.

(c) Kaplan-Meier survival analysis of DFS and OS in TNAC and LK-TNBC according to stage.
